# Tacrolimus: Physicochemical stability challenges, analytical methods, and new formulations

**DOI:** 10.1016/j.ijpx.2024.100285

**Published:** 2024-09-15

**Authors:** Sara Sajjadi, Ali Shayanfar, Farhad Kiafar, Mohammadreza Siahi-Shadbad

**Affiliations:** a Student Research Committee, Tabriz University of Medical Sciences, Tabriz, Iran; b Pharmaceutical Analysis Research Center, Tabriz University of Medical Sciences, Tabriz, Iran; c Department of Pharmaceutics, Faculty of Pharmacy, Tabriz University of Medical Sciences, Tabriz, Iran; d Pharmaceutical and Food Control Department, Faculty of Pharmacy, Tabriz University of Medical Sciences, Tabriz, Iran

**Keywords:** Tacrolimus, Drug stability, Pharmaceutical formulation, Solubility

## Abstract

Tacrolimus, a potent immunosuppressant, is widely used in several formulations to treat organ rejection in transplant patients. However, its physicochemical stability poses significant challenges, including thermal instability, photostability issues, low solubility, and drug-excipient incompatibility. This review article focuses on the details of these challenges and discusses the analytical methods employed to study tacrolimus stability, such as thermal, spectroscopic, and chromatographic methods in different formulations. New formulations to enhance tacrolimus stability are explored, including lipid-based nanocarriers, polymers, and thin film freezing. Researchers and formulators can optimize tacrolimus formulations to improve efficacy and patient outcomes by understanding and addressing these stability challenges.

## Highlights

• Tacrolimus physicochemical stability poses significant challenges.
• Different analytical methods are employed to study tacrolimus stability.
• New formulations of tacrolimus including lipid-based nanocarriers, polymers, and thin film freezing enhance its stability.

## Graphical abstract

**Figure:**
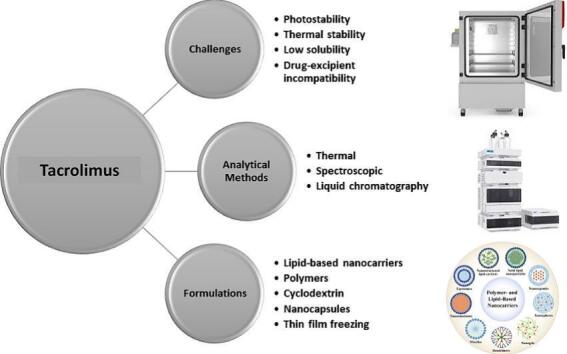

## 1 Introduction

Tacrolimus or FK506 is a macrolide lactone originally extracted from *Streptomyces tsukubaensis* (Yan et al., 2021). It is an immunosuppressant widely used to prevent the rejection of transplantation and treat autoimmune diseases like rheumatoid arthritis (Kaneko et al., 2021; Ong and Gaston, 2021). Tacrolimus was applied to treat psoriasis, vitiligo, and atopic dermatitis (Arora et al., 2020; Liu et al., 2023; Umar et al., 2022). This medicine is also being currently explored as an off-label drug for the treatment of ophthalmic disorders like vernal keratoconjunctivitis (Zhao et al., 2022), atopic keratoconjunctivitis (Hirota et al., 2022), graft-versus-host disease (Lv et al., 2020), corneal graft rejection (Mandal et al., 2023), ocular pemphigoid (Hamed et al., 2023), ocular inflammation, uveitis (Luis et al., 2022), and dry eye disease because of the protective and regenerating of the nervous system effect on the retina (Andrea et al., 2022; Kailasam et al., 2022). It possesses high molecular weight (C_44_H_69_NO_12,_ 804 g/mol) and low solubility (4–12 μg/mL (Ha et al., 2012). The log *P* value of tacrolimus is 2.7 (Fonseca et al., 2021) and its bioavailability is limited (Garg and Garg, 2022). It is classified as class II in the biopharmaceutics classification system which has prominent permeability and low solubility (Radhakrishnan et al., 2021).

Tacrolimus has shown 10–100 times more potency than cyclosporine (Ravanshad et al., 2020). Its binding to a cytosolic protein named FK binding protein (FKBP-12) triggers calcineurin phosphatase inhibition which can dephosphorylate the nuclear factor (NF-kB) in T1 lymphocytes (Annett et al., 2020). Subsequently, the expression of toll-like receptors (TLR) and adhesion molecules, and the release of inflammatory mediators like IL 2 are withdrawn (Llinàs-Mallol et al., 2020). In addition, histamine release from mast cells, expression of inducible nitric oxide synthase, and the synthesis of prostaglandins are suppressed (Albaghdadi et al., 2022). Tacrolimus has the potential to damage β-cells in individuals with type 2 diabetes and metabolic syndrome (Rodriguez-Rodriguez et al., 2021).

Thermolytic, hydrolytic, oxidative, and photolytic tests are stress experiments or forced degradation tests that are mostly used in the pharmaceutical industry (Barrieu et al., 2022). Thermolytic reactions are mainly sensitive to temperature and elevating the temperature can accelerate these reactions under various conditions of relative humidity (RH) in the solid state. Exposure to elevated temperature and different pH values can hasten the hydrolytic reactions (Singh and Narsinghani, 2023). Oxidative degradation of pharmaceuticals is usually the consequence of autoxidation. Initiators such as transition elements, peroxides, or molecular oxygen start the formation of radicals. Photolytic reactions originate from light absorption from exposure to several resources (Zelesky et al., 2023). The starting point of the research on this topic was an article published by our research team about the characterization and determination of the stress tests and degradation products of tacrolimus (Sajjadi et al., 2022).

Stress tests are utilized to predict the degradation pathway and investigate the stability. Drug-excipient compatibility studies are important tests in the preformulation step of pharmaceutical development. It is possible that a drug substance remains stable in its bulk form, but becomes unstable when mixed with other substances in a drug product (Wang et al., 2021b). Drug-excipient interactions influence the chemical, physical, and therapeutic properties and the safety, efficacy, and stability of pharmaceutical ingredients. Physical aspects of drug-excipient incompatibility consist of altering organoleptic properties and delaying the dissolution test and drug degradation is a chemical incompatibility (Patel et al., 2015). Understanding the chemical reactions between drugs and excipients can help to develop a stable formulation. Careful selection of excipients leads to improving the compliance of the patient, promotes bioavailability, and enhances the shelf life (Wang et al., 2021a). Conducting preformulation studies has helped predict and identify potential issues with the active pharmaceutical ingredient (API) before formulating, thereby reducing material wastage and expediting the formulation process (Saatkamp et al., 2023).

Finally, it is important to state that the primary focus was on creating a comprehensive review regarding the stability of tacrolimus. In this review, the current state of research in physicochemical stability challenges of tacrolimus in new formulations is critically examined, focusing on the major findings and challenges faced by the field. The importance of physicochemical stability in advancing our understanding of new formulations is highlighted, shedding light on the potential future directions for research in this area.

## 2 Methods

A structured search was conducted using PubMed, Scopus, and Google Scholar. The keywords including “tacrolimus”, “stability”, “solubility”, and “formulation” were searched in databases. Relevant studies published between 2013 and 2023 that specifically examined tacrolimus formulations were selected for inclusion. Additionally, prior studies published before 2013 were reviewed to enhance the understanding of the subject matter, primarily through reference and citation tracking of the identified articles. Less relevant articles on the stability of tacrolimus formulations were excluded from consideration. The initial screening involved an assessment of the titles and abstracts, followed by a thorough reading of the full texts for final inclusion decisions.

## 3 Physicochemical challenges of tacrolimus

Tacrolimus faces several physicochemical challenges that can impact its stability and efficacy. In this section, some of them are discussed. One such challenge is photostability, as tacrolimus is sensitive to light and can degrade upon exposure. Thermal stability is another concern, as high temperatures can cause degradation of the drug. Additionally, tacrolimus has low aqueous solubility, making it challenging to formulate into aqueous dosage forms like ophthalmic drops. At the end of this section, the incompatibility of the drug-excipient is mentioned.

### 3.1 Photostability

Peterka et al. investigated the photostability of tacrolimus in two manners: solid state and in solution (Rozman Peterka et al., 2019). In a solid-state study, crystalline tacrolimus was dissolved in methanol. Then, the solvent was removed under reduced pressure at 80 °C to obtain a viscous residue which tacrolimus changes to an amorphous form. Photostability test was conducted in various storage conditions which listed in Table 1. The solid-state samples were analyzed with UHPLC after dissolving in acetonitrile: water (70:30).

**Table 1:** Storage conditions of tacrolimus photostability test.

Experimental conditions	Storage conditions	Storage time	
		Solid state	Solution
Artificial sunlight	SUNTEST XLS+, 300–800 nm, 250 W m–2	16 h	2 h
Control (in aluminum foil)		16 h	2 h
Artificial light	Fluorescent lamp with a cool white glow, 2000 lx	22 h	22 h
Control (using aluminum foil)		22 h	22 h

The results of this study proved that tacrolimus was unstable in amorphous form. The most abundant impurity produced under SUNTEST and fluorescent light of solid-state and SUNTEST of the solution was 8-epitacrolimus. The total impurities of the solution under a fluorescent lamp did not reveal a significant difference compared to dark control samples (Rozman Peterka et al., 2019). This research proved that amorphous substances have greater thermodynamic activity and are generally less stable, both chemically and physically, compared to their crystalline counterparts (Peltonen and Strachan, 2020).

Shi et al. developed an HPLC method for analyzing tacrolimus. To study method specificity, they evaluated its ability to separate tacrolimus from degradation products under 4 h of exposure to 4500 Lux light. The study showed that the baseline separation was achieved between tacrolimus and its degradation products (Shi et al., 2012). After these studies, all tacrolimus formulations were kept in light-protected containers (Ezquer-Garin et al., 2017) (Zolgharnein et al., 2024).

### 3.2 Thermal stability

Thermal stability is a critical factor in determining the effectiveness and longevity of pharmaceutical products. In the case of tacrolimus, understanding its thermal behavior is crucial for ensuring its potency and safety. By examining thermal stability studies, valuable insights can be gained into the thermal behavior of tacrolimus and make informed decisions regarding its formulation and storage. Peterka et al. prepared amorphous tacrolimus and the solution for forced degradation studies (Rozman Peterka et al., 2019). They also designed a monthly study for thermal/humidity tests of the amorphous tacrolimus. The test temperature remained constant at 50 °C, but the humidity varied from 30 % to 75 %. Furthermore, the solution was maintained at 60 °C and 25 °C for a day. Thermal degradation of amorphous tacrolimus was associated with an elevation in tacrolimus regioisomer and 8-epitacrolimus. Also, tacrolimus-diene was produced at 50 °C and 75 % RH. Elevating the temperature of the solution (60 °C) raised 8-epitacrolimus as a degradation product. The results indicated that the total degradation product percentages for solid-state tacrolimus at a temperature of 50 °C and relative humidity levels of 30 %, 50 %, and 75 % were 1.37 %, 2.01 %, and 5.81 %, respectively, all of which demonstrate statistically significant differences compared to the initial tacrolimus (0.25 %). For the tacrolimus solution, the quantities of degradation products observed at 60 °C and 25 °C were 0.93 % and 0.25 %, respectively, while the sum of degradation products in the initial solution was 0.26 % (Rozman Peterka et al., 2019). Skytte et al. dissolved tacrolimus in xylene and refluxed at 144 °C for a day. The rearrangement of the allylic ester part of tacrolimus occurred in this study. However, reflux in toluene did not induce the rearrangement reaction due to the lower boiling point (111 °C). So, a high temperature is necessary for this reaction (Skytte et al., 2013).

### 3.3 Solubility

Many factors are involved in the pharmacokinetics and bioavailability of tacrolimus, including substrate for P-gp and CYP3A enzymes and low solubility. Tacrolimus is a highly hydrophobic compound with poor water solubility, posing difficulties in its formulation for oral delivery. Several studies have explored different formulations and strategies to enhance the solubility of tacrolimus, including the use of solubilizing agents, co-solvents, and nanotechnology approaches (Ali et al., 2017; Ponnammal et al., 2018). Some of these techniques are clarified in the following. Overall, understanding the solubility characteristics of tacrolimus is essential for optimizing its pharmacological properties and ensuring its effective use in clinical practice. Further research and development in this area may improve formulations and delivery systems for tacrolimus, ultimately enhancing patient outcomes in transplantation and other immune-related disorders (Patel et al., 2012).

### 3.4 Drug-excipient incompatibility

**Fig. 1:**
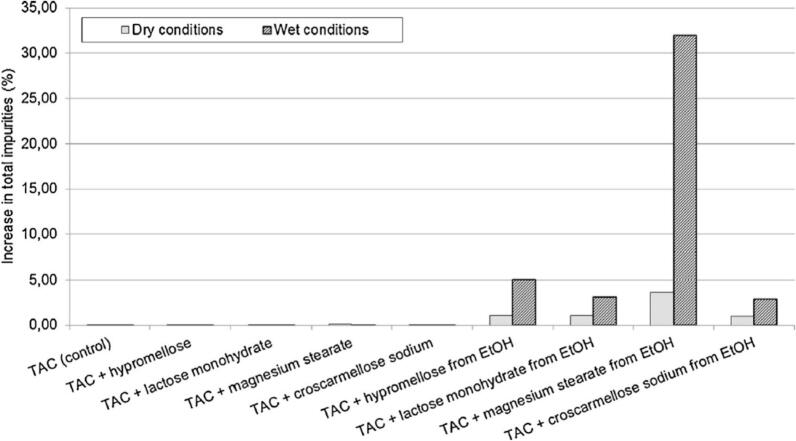
Compatibility test of the physical mixture and ethanolic solution of tacrolimus and excipients under dry (60 °C for a week in sealed vials) and wet conditions (with the addition of 10 μL water; 60 °C for a week in sealed vials) (Peterka et al., 2015). Copyright [2015] [Elsevier].

Drug-excipient incompatibility is a critical factor that must be considered when formulating pharmaceutical products. When drugs and excipients interact in unwanted ways, it can lead to decreased effectiveness of the medication, reduced shelf life, and potentially harmful side effects for the patient. It is essential to thoroughly assess the compatibility of drugs and excipients to ensure the safety and efficacy of the final product. By understanding and addressing potential incompatibilities early in the formulation process, manufacturers can avoid costly recalls and negative health implications for patients. Overall, drug-excipient incompatibility plays a crucial role in ensuring the quality and safety of pharmaceutical products. Peterka et al. investigated the influence of 4 excipients on tacrolimus degradation in the compatibility study (Peterka et al., 2015). For physical compatibility, the binary mixture of tacrolimus with one of the excipients including magnesium stearate, hypromellose, and croscarmellose sodium in the ratio of 1:1 (w:w) was prepared. The mixture of lactose monohydrate and tacrolimus was prepared in a ratio of 20:1. All sample preparation was repeated three times for initial analysis, dry condition (60 °C for a week in sealed vials), and wet condition (with the addition of 10 μL water; 60 °C for a week in sealed vials). Moreover, the compatibility of the ethanolic solution of tacrolimus (60 mg/mL) was analyzed. Consequently, 0.5 L of tacrolimus solution was added to five vials containing magnesium stearate (30 mg), stearic acid (30 mg), hypromellose (30 mg), and croscarmellose sodium (30 mg) and lactose monohydrate (600 mg), respectively. After vortexing the samples for 5 min, they were kept at 40 °C to remove ethanol and analyzed with UHPLC at the beginning, dry condition, and wet condition except for stearic acid that did not have dry condition analysis. The results demonstrated good physical compatibility in wet and dry conditions. However, the increase in total impurities was enhanced in the ethanolic solution. The rationale for using ethanol in this test was that it is used to prepare solid dispersion. In solid dispersion, tacrolimus exists in an amorphous form with more thermodynamic activity than the crystalline form, leading to fast dissolution but low stability. The results suggested that amorphous tacrolimus interacted more with excipients than crystalline form. As shown in Fig. 1, magnesium stearate revealed the fastest degradation among all excipients. However, when stearic acid was present, the increase in total impurities (produced from degradation) was not high. This means that magnesium plays a vital role in the production of degraded products.

**Fig. 2:**
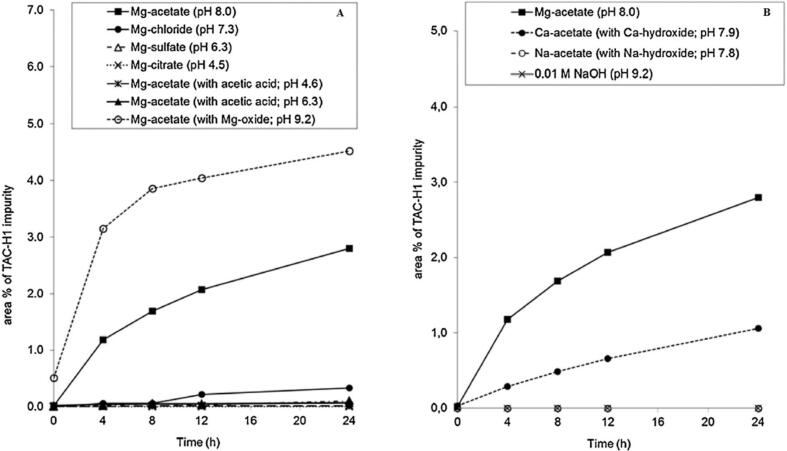
The impact of pH on the rise of impurities in ethanolic solutions of tacrolimus when magnesium ions are present (A) and the increase of impurities in solutions with the presence of magnesium, calcium, and sodium ions under basic pH conditions using 0.01 M NaOH (B) (Peterka et al., 2015). Copyright [2015] [Elsevier].

Furthermore, by analyzing the curves in Fig. 2, it could be understood that this degradation occurs under alkaline conditions (pH > 7). No increase of the impurities was detected without the presence of divalent cations in alkaline conditions. In addition, experiments with monovalent cations like sodium stearate exhibited that the impurities did not increase and divalent cations contributed to the increase in the level of impurities (Peterka et al., 2015). In this research, the increase in total impurities level resulted in degradation and the impurities indicated the contamination from the degraded products.

Regarding drug-excipient incompatibility, it should be noted that there are already several tacrolimus products on the market, but the information available on drug-excipient incompatibility is limited.

## 4 Analytical methods

Various analytical methods were used to evaluate the amount of tacrolimus in the formulation development, solubility analysis, and stability tests. Here, these methods are classified into 3 sections as follow:

### 4.1 Thermal methods of analyses

In recent years, thermal analysis has become increasingly important in the pharmaceutical industry due to its ability to assess the stability of raw materials and finished products. Böer et al. employed thermal analysis and pyrolysis combined with gas chromatography–mass spectrometry (Pyr-GC–MS) to characterize the raw material of tacrolimus. Pyrolysis analysis was conducted under isothermal conditions at 300 °C and 400 °C with GC–MS, with the mass spectrometer set to scan mode to detect ions within the mass range of 25–900 *m*/*z*. The thermal studies utilizing differential scanning calorimetry (DSC), differential thermal analysis (DTA), and DSC-photovisual revealed a desolvation process for the raw materials of tacrolimus. At the same time, dynamic mechanical analysis (DMA) demonstrated the presence of two pseudo-polymorphic forms (monohydrate and sesquihydrate) of tacrolimus. There was a strong correlation between the stoichiometric mass losses observed in TG-dynamical analysis and the identification of product ions in the Pyr-GC/MS technique (Böer et al., 2013).

### 4.2 Spectroscopic techniques

Ultraviolet (UV) spectroscopy plays a crucial role in pharmaceutical analysis due to its ability to accurately and efficiently determine the concentration of a compound in a sample. UV spectroscopy is particularly useful for the analysis of pharmaceuticals because many drugs and compounds absorb UV light at specific wavelengths, allowing for their identification and quantification. By measuring the absorbance of a sample at different wavelengths, pharmaceutical scientists can analyze the purity, concentration, and stability of a drug product. Additionally, UV spectroscopy can be applied to detect impurities, degradation products, and potential interactions within drug formulations, making it a valuable tool in quality control processes within the pharmaceutical industry. In three published articles, tacrolimus was quantified by UV- spectroscopy as can be seen in Table 2. The formulation details of these articles are explained in section 6.1.

**Table 2:** UV spectroscopy of tacrolimus.

Formulation	Wavelength (nm)	Dilution	Ref.
Self-microemulsifying drug delivery system	213	Methanol	(Al-Tamimi and Hussein, 2021)
Solid lipid nanoparticles	294	Phosphate buffer at pH 7.4	(Khan et al., 2022b)
Nanoemulsion	205	Methanol	(Mittal et al., 2021)

### 4.3 Liquid chromatography

High-performance liquid chromatography with ultraviolet detection (HPLC-UV) is a powerful analytical technique commonly used in in-vitro studies to quantify various compounds in a sample. One of the key advantages of using HPLC-UV in in-vitro studies is its high sensitivity and selectivity, which allows researchers to detect and quantify even trace amounts of compounds in complex samples. HPLC-UV also offers excellent reproducibility and precision, making it a reliable tool for quantitative analysis in in-vitro studies. By accurately measuring the concentrations of compounds in a sample, researchers can determine the stability and pharmacokinetics of drugs and assess the potential interactions between different compounds (Mika and Stepnowski, 2016).

Shi et al. developed and validated an HPLC-UV method to determine tacrolimus. They used a C18 column at 60 °C. The mobile phase consisted of acetonitrile-water-phosphoric acid in the ratio of 700: 300: 0.2 and flowed at the rate of 1 mL/min. 20 μL of tacrolimus-containing sample was detected at a wavelength of 215 nm. This method had a good linearity (r^2^ = 0.9992) in the range of 10.20 to 100.20 g/mL. The accuracy range of tacrolimus was between 98.53 % and 99.57 % and the precision was less than 3.5 %. The limit of detection (LOD) and limit of quantification (LOQ) were measured at 0.128 and 10.20 g/mL, respectively (Shi et al., 2012).

Namiki et al. determined tacrolimus by reverse phase HPLC-UV method. Tacrolimus was quantified by a system containing a C18 column at 50 °C, water: isopropyl alcohol: tetrahydrofuran (5: 2: 2) as a mobile phase with the flow rate of 0.8 mL/min, and a UV detector (at 220 nm). The validation parameters demonstrated good linearity (r^2^ = 0.9999), acceptable repeatability (RSD <1 %), and suitable accuracy (recovery range of 99.3 % to 101.0 %) (Namiki et al., 1995).

Ponnammal et al. prepared disintegrating tablets of amorphous solid dispersion of tacrolimus. They obtained the amorphous tacrolimus from hot melt extrusion of different polymers, Soluplus, polyvinylpyrrolidone vinyl acetate, and hydroxypropyl cellulose. The solid dispersion samples were stored at 40 °C /75 % RH for 3 months. At last, the HPLC-UV method was used to measure drug content. The results showed that tacrolimus content was decreased by changing the polymer from polyvinylpyrrolidone vinyl acetate to hydroxypropyl cellulose and Soluplus. The XRD patterns exhibited an amorphous structure in tacrolimus samples with all polymers extruded. These stable amorphous solid dispersions displayed a better dissolution rate (Ponnammal et al., 2018).

Hirasawa et al. formulated tacrolimus in a universal orbicular vehicle. It was created through the gelling of a solution containing tacrolimus, starch syrup, gelatin, and triethyl citrate. The stability studies were carried out in two modes: sealed with a film and unsealed. The samples were placed in Petri dishes at several storage conditions and light exposure (1.2 million lux). Data obtained from HPLC-UV displayed an obvious degradation in tacrolimus by light exposure (21 %) compared to other stability tests (Hirasawa et al., 2020).

Kovačević et al. developed a tacrolimus-loaded nanostructured lipid carrier as clarified in section 6.1.3. Tacrolimus in the mixture containing lipid was determined by the HPLC-UV as illustrated in Table 3 (Kovačević et al., 2020). Sahakijpijarn et al. minimized the excipients of inhalable tacrolimus through thin film freezing. Tacrolimus quantification was done with HPLC-UV (Sahakijpijarn et al., 2020). Rebibo et al. worked on amazing research about the use of nanocapsules containing tacrolimus for ophthalmic administration. Tacrolimus content was measured with an HPLC-UV system (Rebibo et al., 2021). Table 3 illustrates HPLC-UV analytical method conditions in these formulations.

**Table 3:** The HPLC-UV conditions of tacrolimus quantification.

Formulation	Column	Mobile phase	Flow rate (mL/min)	Wavelength (nm)	linearity range	Ref.
Tacrolimus disintegrating tablets	C18 at 40 °C	Acetonitrile: Methanol: Water (45:45:10)	1.5	214	0–100 μg/mL	(Ponnammal et al., 2018)
Tacrolimus in universal orbicular vehicle	C18 at 50 °C	Water: tetrahydrofuran: 2–Propanol (5:2:2)	0.7	220	1–100 μg/mL	(Hirasawa et al., 2020)
Tacrolimus-nanostructured lipid carrier	C18 at 50 °C	Acetonitrile: Water: Isopropanol (70:28:2)	1	210	3–27 μg/mL	(Kovačević et al., 2020)
Thin film freezing tacrolimus	C18 at 50 °C	A: 0.2 % phosphoric acid B: acetonitrile	1.5	215	1–250 μg/mL	(Sahakijpijarn et al., 2020)
Tacrolimus-loaded nanocapsules	C18 at 60 °C	Acetonitrile: water (95:5 *v*/v)	0.5	213	0–200 μg/mL	(Rebibo et al., 2021)

LC-MS/MS is an essential tool in in-vivo studies of immunosuppressants due to its high sensitivity and specificity in quantifying the drug levels in biological samples (Oellerich et al., 1998). Tacrolimus has a narrow therapeutic window, making accurate measurements crucial for dosing adjustments. LC-MS/MS can accurately measure tacrolimus levels in blood, plasma, and tissue samples, allowing researchers to monitor drug concentrations over time and optimize dosing regimens for individual patients (Sallustio, 2010). Additionally, LC-MS/MS can also detect metabolites and breakdown products of tacrolimus, providing insight into its pharmacokinetics and potential interactions with other drugs. Overall, the precision and reliability of LC-MS/MS make it an indispensable tool in understanding the pharmacodynamics and pharmacokinetics of tacrolimus in in-vivo studies (Andrews et al., 2017). This section covers the conditions of the LC-MS/MS analytical method to determine the amount of tacrolimus in biological samples in four important formulations. The experimental conditions are displayed in Table 4.

**Table 4:** The LC-MS/MS conditions of tacrolimus quantification.

Formulation	Column	Mobile phase	Flow rate (mL/min)	MS/MS parameters⁎	linearity range	Ref.
Tacrolimus self-microemulsifying drug delivery system	C18	Acetonitrile: water (90:10 v/v)	0.3	Triple quadrupole detector, Positive electrospray ionization (ESI^+^), selected reaction monitoring: 826.6 → 616.4 m/z	0.5–25 ng/mL	(Tao et al., 2020)
Tacrolimus in cyclodextrin solution	C18	Ammonium acetate (pH = 5.5)	0.5	Quadrupole Time of Flight (QTOF MS), ESI^+^, capillary voltage = 3.2 kV, source temperature: 120 °C, cone gas flow: 50 L/h, desolvation temperature and flow: 400 °C, 800 L/h, 50–1000 *m*/*z*	not available	(Prajapati et al., 2020)
Polymeric ocular formulation of tacrolimus	C8	A: 0.1 % v/v formic acid in water B: 0.1 % v/v formic acid in acetonitrile	0.5	ESI^+^, multiple reaction monitoring: 826.3 → 415.2 m/z	62.5–1000 μg/mL	(Badr et al., 2021)
Tacrolimus in the dried matrix on paper discs	C18	A: 2 mmol/L ammonium acetate in water, 0.1 % formic acid B: 2 mmol/L ammonium acetate in methanol, 0.1 % formic acid	0.5	ESI^+^, capillary voltage = 2.8 kV, cone voltage: 20 V, desolvation temperature and flow: 550 °C, N_2_ 800 L/h,cone gas: 50 L/h N_2_, collision gas: 0.15 mL/min Ar	1.1–30.8 ng/mL	(Bressán et al., 2021)

⁎ The parameters were added here based on the mentioned ones in the cited references

In a study about self-microemulsifying drug delivery systems, LC-MS/MS was utilized to determine the concentration of tacrolimus in blood (Tao et al., 2020). Prajapati et al. applied LC-MS/MS to investigate the rate of degradation of tacrolimus in cyclodextrin solutions (Prajapati et al., 2020). In addition, Badr et al. used this method to quantify tacrolimus in eye tissue. The LOD and LOQ were 13.46 μg/mL and 40.80 μg/mL, respectively (Badr et al., 2021). Tacrolimus was monitored by positive electrospray ionization in these surveys.

Bressán et al. validated an LC-MS/MS as the preferred method for measuring immunosuppressants in therapeutic drug monitoring. They also considered dried blood spots (DBS) as a viable alternative for sample collection in this context. This method showed good repeatability (RSD <15 %) and accuracy (98.6 % - 99.6 %) (Bressán et al., 2021).

## 5 The enhanced efficacy due to improved solubility or stability

The enhanced efficacy of pharmaceutical products often correlates directly with their solubility and stability, two critical factors that influence their bioavailability and therapeutic effectiveness (Zheng and McClements, 2020).

Improved solubility enhances a drug's ability to dissolve in biological fluids, thus increasing its absorption rate in the gastrointestinal tract and facilitating a quicker onset of action. For instance, many lipophilic drugs face challenges in solubilization, which can significantly limit their systemic circulation and efficacy. Innovations such as the development of nanosuspensions, solid dispersions, or the use of surfactants and co-solvents have led to substantial improvements in solubility (Bhalani et al., 2022). This allows for lower dosages to achieve desired therapeutic effects, reducing potential side effects and improving patient compliance (Chettri et al., 2024).

Stability, on the other hand, refers to the drug's ability to maintain its chemical integrity and physical properties over time. Improved stability prevents degradation due to environmental factors such as moisture, light, or heat, which can compromise a drug's efficacy that can decrease the effectiveness or can cause adverse effects. Techniques such as encapsulation, lyophilization, and the incorporation of stabilizing agents can shield active ingredients from these factors, thus preserving their therapeutic benefits (Khan et al., 2022a). A stable formulation can also extend the shelf-life of a pharmaceutical product, ensuring that patients have access to effective treatments over time. Furthermore, stable formulations promote uniform distribution and improved absorption of the drug (Kamelnia et al., 2024). Conversely, unstable formulations can result in diminished drug quality, leading to reduced active ingredient levels and efficacy (Elumalai et al., 2024). Stable formulation ensures evenly distribution and improved absorption. Consequently, it is essential for tacrolimus dosage forms to retain the necessary quality attributes throughout their shelf life to prevent potential safety and efficacy concerns associated with tacrolimus (Rahman et al., 2013).

## 6 Novel formulations

Nanoscale drug delivery systems including nanomicelles, nanoliposomes, nanoemulsions, and polymeric nanoparticles demonstrated better actions compared to conventional formulations. Nanocarriers have great features like controlling the release of drugs and perfect crossing through lipophilic barriers.

### 6.1 Lipid-based nanocarriers

#### 6.1.1 Self-microemulsifying drug delivery systems (SMEDDS)

SMEDDS can increase the ability of hydrophobic drugs to dissolve in an aqueous solution and prevent recrystallization during preparation and storage (Jadhav Bharat et al., 2024). It is mainly consisting of oil, surfactant, and co-surfactant (Mansoori, 2023). The SMEDDS assembles into nano oil-in-water (o/w) emulsion droplets upon gastrointestinal liquids. Its absorption can be improved by inhibiting *p*-glycoproteins, micelles formation, and lymphatic transport (Yan et al., 2020).

**Fig. 3:**
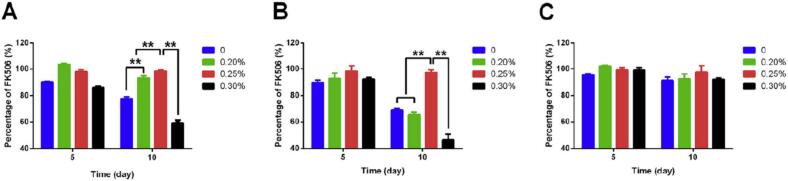
The effect of citric acid on the degradation of tacrolimus in SMEDDS under thermal (40 ± 2 °C) (A), humidity (90 ± 5 %) (B), and radiation (4500 ± 500 lx) (C) (Tao et al., 2020). Copyright [2020] [Elsevier].

In a study, the effects of temperature, humidity, radiation, pH, and dilution on the physicochemical stability of SMEDDS were investigated. Tacrolimus-SMEDDS was prepared by mixing ethyl oleate, Solutol, and Transcutol® as oil, surfactant, and co-surfactant, respectively. Then it was placed in glass vials under three different stress conditions: i) temperature: 40 ± 2 °C, ii) RH: 90 ± 5 %, iii) radiation: 4500 ± 500 Lux, for 10 days. To evaluate the effect of pH on the stability of the formulations, 0.2, 0.25, and 0.3 % citric acid was added to the samples. In the dilution test, the drug amount, polydispersity index (PDI), and size were analyzed at 8 dilution ratios containing 1:100, 1:200, 1:300, 1:400, 1:1000, 1:2000, 1:3000, and 1:5000. The results demonstrated that the amount of tacrolimus decreased to 77 % after 10 days of thermal test and adding 0.25 % citric acid increased it to about 98 %. While in more than 0.25 % citric acid, the amount of tacrolimus was reduced (Fig. 3A). In the humidity test, tacrolimus was decreased to 68 % and improved to 97 % when the optimum quantity of citric acid (0.25 %) was added (Fig. 3B). Tacrolimus-SMEDDS with and without 0.25 % citric acid displayed high drug amounts with 91 % and 97 % of tacrolimus remaining at day 10 (Fig. 3C). The findings indicated that the pH value of the SMEDDS was a significant factor influencing drug degradation of tacrolimus compared to temperature. The pH of the formulation was decreased to 5 (mild acid) after adding 0.25 % citric acid and promoting its stability during storage.

**Fig. 4:**
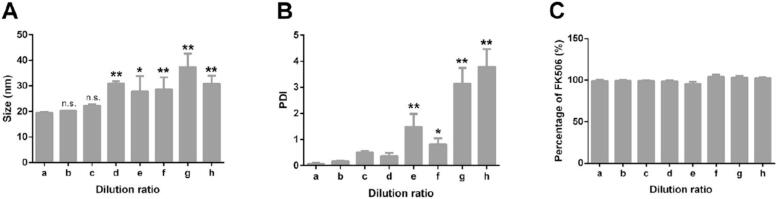
The effect of dilution on size (A), PDI (B), and amount of drug (C) of the tacrolimus-SMEDDS. The dilution ratios comprised 1: 100 (a), 1: 200 (b), 1: 300 (c), 1: 400 (d), 1: 1000 (e), 1: 2000 (f), 1: 3000 (g), and 1: 5000 (h) (Tao et al., 2020). Copyright [2020] [Elsevier].

In the formulations with dilution above 1:200, the size and PDI were maintained at 20 nm and 0.1, respectively. However, the size and PDI of formulations with dilution ratios between 1:300 to 1:5000 were increased up to 40 nm and 4, respectively (Fig. 4A and B). The emulsion's size was dependent on the quantity of surfactant used. Distributing less amount of surfactant leads to aggregation and increasing the size. The results proved that all formulations contained more than 98 % tacrolimus (Fig. 4C). Thus, the stability of tacrolimus in the SMEDDS was not impacted by the dilution (Tao et al., 2020).

Al-Tamimi et al. prepared another SMEDDS containing Maisine® CC, Labrasol® ALF, and Transcutol® as oil, surfactant, and co-surfactant, respectively. Thermodynamic stability tests including the heating-cooling cycle (6 cycles of 48 h at 4 and 45 °C), centrifugation (3500 rpm for 30 min), and freeze-thaw cycle (3 cycles of 48 h at −21 and + 25 °C) were examined. To predict the robustness of formulations to dilution and the effect of pH on stability, the samples were diluted with water and 0.1 N HCl in different ratios of 1:50, 1:100, 1:250, and 1:1000 and were placed at room temperature for 24 h. To assess the quantity of tacrolimus in the formulations, UV–visible spectroscopy was used at 213 nm. The outcomes of the study illustrated that this formulation had good thermodynamic stability and resiliency to pH changes probably due to its non-ionic nature (Al-Tamimi and Hussein, 2021).

The incorporation of tacrolimus into topical formulation maximized the local absorption and enhanced the safety and efficacy of tacrolimus (Gomes et al., 2023). The hybrid polymer and lipid system possesses advantages such as great stability, high biocompatibility, control of the drug release, and decreased immunogenicity. These formulations could bind to the skin through their positive surface charge (Hazari et al., 2023). Fereig et al. prepared a topical tacrolimus formulation in a hybrid nanoparticle of chitosan and lecithin for psoriasis to avoid side effects of systematic treatment. The prepared formulations were different in terms of chitosan solutions (0.5, 1, and 2.5 %), lecithin: chitosan ratios (5:1, 10:1, and 20:1), and the type of co-solvent (Tween 80 and olive oil).

The stability test was performed at 4 °C for 12 weeks and particle size, PDI, zeta potential, and entrapment efficiency (EE %; Eq. (1)) were assessed.

(1) $$\mathrm{EE}\left(\%\right)=\frac{\left(\mathrm{Total amount of tacrolimus}-\mathrm{free tacrolimus}\right)}{\mathrm{Total amount of tacrolimus}}$$

The findings indicated that reducing the chitosan concentration led to the formation of smaller particles with a high EE%. Although higher lecithin to chitosan ratio resulted in smaller particle sizes and increased zeta potential, it notably reduced the EE%. Formulations made with Tween 80 produced smaller particles compared to those made with olive oil. The formulation containing Tween 80, 0.5 % chitosan, and a lecithin-to-chitosan ratio of 10:1 showed significant drug release. Therefore, the optimum formulation was prepared with 0.5 % chitosan solution, lecithin (in the ratio of 10:1 lecithin: chitosan), and Tween 80 as a co-solvent.

The results confirmed that EE % and zeta potential decreased by 15.1 % and 5.7 mV, respectively and PDI and particle size increased by 0.55 and 154.36 nm, respectively. The data proposed agglomeration occurrence during storage time and the researchers suggested lyophilizing the formulation (Fereig et al., 2021a).

Gote et al. formulated a nanomicelle of self-assembling tacrolimus to treat age-related macular degeneration. Design of the experiment was utilized for nanomicelles preparations which had three levels of three independent variables including the time of sonication (25, 22.5, and 20 min), PEG‑hydrogenated castor oil-40 (3.5, 2, and 0.5 %), and octyxonyl-40 (3, 2, and 1 %). The responses of the experiment were hydrodynamic size, EE %, zeta potential, PDI, and loading efficiency (LE %). The formulations were prepared by dissolving PEG‑hydrogenated castor oil-40, octyxonyl-40, and tacrolimus in ethanol (above the critical micellar concentration of the polymers). The solvent was subsequently evaporated to create a thin film of tacrolimus and polymers. The film was then rehydrated with water, sonicated, filtered through a 0.22 μm filter, and stored at 4 °C. The optimum formulation based on the design of the experiment had 3.5 % PEG‑hydrogenated castor oil-40 and 1 % octyxonyl-40. Its sonication time was 20 min. The formulation resulted in lower PDI, zeta potential, and size, and higher EE % and LE %. This formulation was selected for the dilution test. Seven dilution factors were examined in this study (10, 20, 40, 50, 100, 150, and 200). The results verified that dilution up to 200 times did not have a meaningful effect on the size and slightly changed the zeta potential and PDI. This indicated the stability of formulation in aqueous state (Gote et al., 2020).

Conventional self-emulsifying systems developed with a liquid phase can have certain drawbacks, including the precipitation of bioactive molecules in vivo, challenges in product handling, and limited lymphatic transport. These issues further constrain their broader use in applications (Rajpoot et al., 2020).

#### 6.1.2 Solid lipid nanoparticle (SLN)

SLNs are promising drug delivery systems that offer plentiful advantages over traditional drug formulations. SLNs offer several benefits, including protection of drugs from harsh environmental conditions, the ability to be produced on a large scale through high-pressure homogenization, as well as their biocompatibility and biodegradability (Ghasemiyeh and Mohammadi-Samani, 2018). Three different types of SLNs can be utilized: the homogeneous matrix model, drug-enriched shell model, and drug-enriched core model (Alsaad et al., 2020). In the homogeneous matrix model, the drug is dispersed throughout the lipid matrix, providing sustained drug release over time. The drug-enriched shell model involves a lipid shell that surrounds a drug core, allowing for a controlled release system. Finally, the drug-enriched core model features a drug core surrounded by a lipid matrix, facilitating precise delivery to specific areas of the body. Each of these SLN models offers unique benefits that can be customized to suit the requirements of different drugs and therapeutic applications (K, 2018).

Khan et al. formulated an SLN gel of tacrolimus. The SLNs were composed of 0.1 g tacrolimus, 1 mg stearic acid, 10 mL ethanol, three amounts of Tween 80 (1, 1.25, and 1.5 mg), and Span 80 (0, 0.25, and 0.5 mg), and up to 100 mg water. One of the formulations had 0.0015 mg chitosan for controlling the release and boosting the EE %. The gels consisted of three components: 1 g sodium alginate, 5 g glycerol, and 1 g triethanolamine. The physicochemical properties of the SLNs were assessed by evaluating the size, zeta potential, PDI, and EE %. The formulations without Span 80 as a second surfactant had a low EE %. The resulting data proved that the combination of two surfactants (Span 80 and Tween 80 with HLB values of 4.3 and 15, respectively) was ascribed to the stability of nanocarriers. Moreover, the presence of chitosan led to a higher viscosity in the formula, thereby the SLNs achieved the optimum skin permeation and retention properties (Khan et al., 2022b).

Sun et al. provided tacrolimus-SLNs in-situ gel. It was created using a combination of homogenization and ultra-probe sonication. Initially, Compritol® 888 ATO and glyceryl monostearate were dissolved in dichloromethane, heated, and tacrolimus was added until a transparent drug-lipid mixture was formed. The organic solvent was removed through evaporation and the lipid film was dried further to ensure all solvent was eliminated. Simultaneously, an aqueous phase containing Tween-80 and glycerin was heated and added to the drug-lipid mixture to create a pre-mixture. This mixture was then homogenized using an ultra-sonicator to form tacrolimus SLNs. Finally, the gel was obtained by combining tacrolimus SLNs with a poloxamer solution in an ice bath. The samples were kept at 40 °C/ 75 % RH for three months and EE %, zeta potential, PDI, and size were measured on months 0, 1, and 3. At the end of the test, the samples showed good stability and the viscosity of the formulation altered negligibly (Sun and Hu, 2021).

Jain and colleagues carried out research concentrating on creating, refining, and defining new lipid-based nanoformulations designed for delivering tacrolimus topically. Liquid crystalline nanoparticles (LCNP), SLN, liposomes, and NLC loaded with tacrolimus were created using selected lipids and surfactants based on their emulsification properties. These formulations were optimized to enhance EE, reduce particle size and PDI, and attain a desirable zeta potential before being incorporated into gels. The gels containing the nanoformulations underwent analysis for rheology, viscosity, in-vitro drug release, and skin permeation. In-vivo experiments using a mouse tail model and skin irritation tests were carried out, including measurement of transepidermal water loss. The developed nanoformulations exhibited optimal characteristics such as particle size (<200 nm), PDI (<0.3), zeta potential (≥ − 10 mV), and high EE (>85 %). These formulations demonstrated enhanced penetration of tacrolimus into the skin, with Tac-LCNP, Tac-SLN, Tac-NLC, and Tac-liposomes loaded gels displaying significantly superior dermal absorption compared to free tacrolimus gel and Tacroz™ Forte. While Tac-liposomes showed lower dermal bioavailability than Tacroz™ Forte, they still exhibited improved skin penetration without causing substantial irritation. In conclusion, the developed nanoformulations achieved enhanced skin penetration of tacrolimus in comparison to the commercially available Tacroz™ Forte ointment (Jain et al., 2019).

Kang et al. aimed to create and assess tacrolimus-SLNs with thermosensitive properties for improved penetration and retention. In this study, tacrolimus-SLNs were created using a modified emulsification and low-temperature solidification process. The drug-oil and lipid phases were produced independently and combined at 5000 rpm and 50 °C for 20 min, then carefully added to an aqueous phase at 70 °C for 15 min. The resulting mixture was further mixed at 15000 rpm and 70 °C for 20 min before being rapidly cooled in ice water to form the tacrolimus-SLN emulsion. Sonication was used to create a stable tacrolimus-SLN suspension in a chiller at 0 °C. The drug-oil phase was prepared by dissolving cocoglyceride and tacrolimus in methylene chloride, while the lipid phase was obtained by dissolving Brij® 93 and soybean lecithin in ethanol. The aqueous phase consisted of 2 % surfactant (Poloxamer 188 or Brij® 58) in distilled water. The researchers successfully produced tacrolimus-loaded thermosensitive nanoparticles with various surfactants on the particle shell to enhance skin permeation and distribution. The tacrolimus-SLNs were found to contain tacrolimus in an amorphous state with high loading efficiency. Ex-vivo and in-vivo evaluations demonstrated that tacrolimus-SLNs penetrated extremely into the skin compared to the reference product (Protopic®), as confirmed by FT-IR patterns showing drug distribution in deeper skin layers (Kang et al., 2019).

SLNs come with a few drawbacks as well. Their ideal crystalline structure results in low drug loading efficiency and can lead to drug expulsion during crystallization under storage conditions. Additionally, these formulations often experience an initial burst release (Ghasemiyeh and Mohammadi-Samani, 2018).

#### 6.1.3 Nanostructured lipid carriers (NLC)

NLCs possess a greater capacity for drug loading due to their non-ideal crystal structure, allowing them to prevent drug loss by minimizing lipid crystallization during production and storage. The incorporation of liquid lipids in NLC formulations helps to reduce the expulsion of the loaded drug both post-formulation and throughout storage. Additionally, NLCs can enhance drug solubility within the lipid matrix and exhibit more controlled release profiles in comparison to SLNs(Ghasemiyeh and Mohammadi-Samani, 2018).

Kovačević et al. formulated a nanostructured lipid carrier of tacrolimus to improve drug delivery. First, 20 lipid excipients from 6 groups of lipids (waxes, monoglycerides, diglycerides, triglycerides, fatty acids, and liquid lipids) were chosen to find the most suitable one with high encapsulation efficacy. Then, 5 stabilizers were used to produce lipid nanoparticles. Tacrolimus-loaded lipid nanoparticles were produced by dissolving tacrolimus in the melted lipid mixture at 75 °C. At last, the physicochemical stability was identified and biopharmaceutical efficacy was evaluated. There was only a slight variance in the polar and hydrogen bonding energies between tacrolimus and monoglycerides. The greatest variations were observed in waxes, indicating no significant interaction between waxes and tacrolimus. The euclidean distances between tacrolimus and monoglycerides were the smallest and the miscibility between them was the best making monoglycerides the most suitable excipients for NLC. Compritol® 888 CG and Imwitor® 900P were chosen as solid lipids for further study due to their elevated melting temperature. The stability results emphasized tacrolimus was stable under applied production circumstances. Examination of the performance of nonionic polyhydroxy surfactants showed that Plantacare® 810, the stabilizer with short alkyl chain, high critical micelle concentration (CMC), and low molecular weight, exhibited the most favorable characteristics for particle stabilization. The system remained physically stable at 25 °C for 30 days. The research conducted suggested that polyhydroxy surfactants derived from natural sources could potentially serve as a novel group of excipients for enhancing the stability of NLC (Kovačević et al., 2020).

Savić et al. progressed tacrolimus dermal delivery by lecithin-based nanoemulsion (NE) and NLC with glyceryl palmitostearate (Precirol® ATO 5) as solid lipid and propylene glycol monocaprylate, type II (Capryol™ 90) as liquid lipid via hot high-pressure homogenization method. Eight formulations were designed as listed in Table 5 and their physiochemical properties were characterized during 6 months of storage at room temperature.

**Table 5:** Composition of NLC and NE formulation of tacrolimus.

Formulation	Lecithin	Precirol® ATO 5	Capryol® 90	Butylated hydroxytoluene	Polysorbate 80	Water
NLC1	1	6	4	0.05	1	87.95
NLC2	1	5	5	0.05	1	87.95
NLC3	1	4	6	0.05	1	87.95
NLC4	1	2	3	0.05	1	92.95
NLC5	2	4	6	0.05	2	85.95
NLC6	0.5	4	6	0.05	0.5	88.95
NLC7	1	4	6	0.05	–	88.95
NE	1	–	10	0.05	1	87.95

Studying placebo NLC formulations with equal lipid content (10 %) but varying solid/liquid lipid ratios (NLC1, NLC2, NLC3) confirmed that a greater proportion of solid/liquid led to increased particle size. The particle size and PDI of NLC3 did not change noticeably over six months of storage, whereas testing of NLC1 and NLC2 was stopped after one month because those samples had gelled and their structure changed. When looking at two formulations with equal proportions of solid and liquid lipids but varying overall lipid quantities (10 % in NLC3 compared to 5 % in NLC4), it was noted that the particle size was notably larger in the formulation with higher lipid content. While NLC4 remained stable for six months during storage, a slight yellowish tint was noticed after three months. The acceptable reason for this color change could be correlated with the oxidation of butylated hydroxytoluene (BHT). Because all formulations contained the same amount of BHT, the higher BHT in lipid particles with less lipid content made the oxidation more noticeable in NLC4 compared to NLC3. Variations in surfactant levels in formulations did not show a noteworthy impact on particle size as strongly as the lipid phase content. When comparing the size of particles and the PDI for placebo NLC and NE with a 10 % lipid phase, it was noted that the droplets in NE were fairly smaller. In the fourth month, the separation of oil droplets in NE was recognizable. This could be explained by the utilization of Precirol® ATO 5 and the solid lipid matrix in place of oil droplets which appear to improve the stability of NLC3. None of the parameters exert a major influence on the zeta potential. The formulation with a low amount of Capryol™ 90 (NLC4) had a high pH value (4.77), while the formulation with a high Capryol™ 90 (NE) had the lowest pH value (4.22). Formulation NLC3 had the highest conductivity among the formulations because it contained the highest amount of lecithin, while NLC1 had the lowest conductivity due to its high viscosity. As the formulations were kept, a decrease in pH levels and a rise in conductivity were noted for all formulations, possibly caused by the hydrolysis of lecithin. Formulation NLC1, which had a high proportion of solid to liquid lipid, exhibited significantly high viscosity. As the solid-to-liquid lipid ratio was decreased, the viscosity decreased accordingly. When comparing formulations with the equal 4:6 solid-to-liquid lipid ratio but varying total lipid phase amounts (10 % in NLC3 and 5 % in NLC4), it was observed that decreasing the total lipid phase reduced the viscosity values. Comparing formulations with varying amounts of surfactants, the higher levels of lecithin and polysorbate 80 in NLC5 resulted in an increased viscosity compared to NLC3. However, having a decreased amount of lecithin and polysorbate 80 (NLC6) or no polysorbate 80 (NLC7) did not have a significant impact on the viscosity of the formulations. This may be correlated with the lower concentration of surfactants in the water phase of these formulations because they are primarily located at the lipid-water interface. Overall, it can be recommended that developed NLC3 showed more capability to utilize as a carrier for tacrolimus when formulated into a lotion for dermal delivery (Savić et al., 2019).

Viegas et al. created NLCs using a hot homogenization technique, where a pre-emulsion was formed by mixing an aqueous phase and oil phase at 10 °C above the solid lipid's melting point. The aqueous phase contained polyethyleneimine, poloxamer 407, and phosphate buffer, while the oil phase included glycerol di-stearate and oleic acid. Tacrolimus was added to the oil phase. The pre-emulsion was sonicated and then siRNA was added before incubation at room temperature. A control NLC without tacrolimus and siRNA was also prepared. After physicochemical characterization, the physical stability of both NLC formulations was determined over 30 days at different temperatures (4, 25, and 37 °C). The findings indicated that the NLCs (control and tacrolimus variants) remained stable at 25 °C and 37 °C throughout the study. However, when stored at 4 °C, the NLCs displayed significant instability regardless of the presence of the drug. It was observed that the addition of tacrolimus did not impact the stability of the nanostructured system. The study developed multifunctional NLCs with high encapsulation efficiency, small particle size of tacrolimus, and effective complexation with TNF-α siRNA. The particle size of the NLCs was around 230 nm, allowing for the distribution of drugs to deeper layers of the skin. The positive zeta potential of the NLCs containing tacrolimus could be attributed to the presence of the positively charged polymer PEI on the particle surface, which enhanced cellular transfection efficiency. The study also found that the NLCs were physically stable when stored at room temperature but showed an increase in particle size when stored at 4 °C. The study on release demonstrated that tacrolimus was released in a controlled manner, and how it entered and stayed in the skin tissue appeared favorable for use in topical applications. Overall, the NLCs showed promising characteristics for drug delivery applications (Viegas et al., 2020).

Dasineh et al. prepared drug-loaded chitosan-coated NLCs (CCNLCs) to address certain limitations. The study involved the preparation and optimization of tacrolimus-loaded NLCs using the solvent displacement technique. Solid lipids such as glyceryl monostearate and stearic acid were utilized, along with liquid lipids like oleic acid and stabilizers like Tween 80. Characterization of the nanoparticles was done through zeta potential and size measurements, drug loading (DL) determinations, scanning electron microscope (SEM), and FT-IR analysis. The release profile of tacrolimus from both NLCs and CCNLCs was examined, with results showing the impact of several parameters on particle size. The particle sizes of the formulations were less than 100 nm, and the change in zeta potential from negative to positive in CCNLCs was likely due to the chitosan coating. SEM images confirmed small dimensions and FTIR analysis revealed no undesired chemical reactions. Both NLCs and CCNLCs exhibited sustained release patterns, with chitosan-coating resulting in a more extended release and reduced burst effect. The study demonstrated the successful tacrolimus-loaded NLCs formation and their chitosan coating, showing promising characteristics for addressing bioavailability issues associated with conventional tacrolimus formulations (Dasineh et al., 2021).

Furthermore, the addition of additional materials, processes, and energy for the creation of formulations within the NLC system unavoidably raises production expenses, resulting in increased prices for the products. The use of NLCs is also constrained by the types of materials employed in their formulation. For example, polysorbate 80 (Tween 80) can only be used as a stabilizer in preparations designed for adult patients due to its potential to cause skin irritation in children. Since NLC systems are generally oil-in-water emulsions, it presents difficulties in formulating drug-loaded NLCs into solid dosage forms (Wathoni et al., 2024).

#### 6.1.4 Nanoemulsion (NE)

NEs are lipid-based carrier systems comprising oil, surfactant, and co-surfactant (Soni and Sharma, 2021). Nanoemulsions offer several benefits, making them effective transport systems for various applications. They exhibit a larger surface area and increased free energy, which enhances their stability by avoiding issues like creaming, flocculation, coalescence, and sedimentation. These formulations can be designed as foams, creams, liquids, or sprays. They are safe and non-irritating, allowing for easy application on skin and mucous membranes, and can be taken orally if they contain biocompatible surfactants. Nanoemulsions do not harm healthy human cells, making them suitable for therapeutic uses in humans. They improve the absorption of oil-soluble supplements in cell cultures, supporting cell growth and facilitating toxicity studies for oil-soluble drugs. Additionally, nanoemulsions can serve as alternatives to liposomes and vesicles, and their small size allows for better penetration through the skin, enhancing the delivery of active ingredients (Malode et al., 2021). Several significant benefits associated with nanoemulsion characteristics have been highlighted over the past decade. These include effective drug release at the right rate, extended effectiveness, regulation of drug absorption, minimal side effects, and the ability to protect drugs from enzymatic or oxidative degradation (Nishitani Yukuyama et al., 2017).

Mittal et al. examined a tacrolimus-loaded NE gel to enhance its anti-psoriatic effects. Oils with strong anti-inflammatory properties were chosen. Specific surfactants (Solutol, Unitop FFT-40, Tween 20, and Tween 80) and co-surfactants (Transcutol P, Propylene glycol, PEG 400, and Lauroglycol 90,) were tested for solubility and miscibility with oils, and different ratios of surfactant and co-surfactant (1:0, 1:1,1:2, 1:3, 2:1, 3:1, 4:1, and 5:1) were evaluated to determine the optimal combination for constructing NEs. The physical stability of the NE was estimated by subjecting it to three freeze-thaw cycles at −20 °C for 24 h each, centrifugation studies at 5000 rpm for 30 min, and six heating-cooling cycles between 4 °C and 40 °C. To prepare an NE gel, Carbopol-934 was dispersed in the NE solution with the help of sieving and continuous stirring, followed by the addition of triethanolamine to form the gel. The selection of oils for NE preparation was focused on fish oil and linseed oil, both rich in omega-3 fatty acids (docosahexaenoic acid and eicosapentaenoic acid) for their combined anti-inflammatory effects. The surfactant, Tween 80, was chosen based on solubility studies and its widespread use in topical NEs. Co-surfactant selection, Transcutol P, was based on its compatibility with both oils and Tween 80, as well as its role in reducing interfacial tension and aiding in the solubilization process. The ratio of 4:1 was chosen based on the pseudo-ternary phase diagram, indicating optimal NE formation. The criteria chosen to obtain the optimized NE included a small hydrodynamic diameter for improved drug permeation and a smaller PDI. The physical stability testing was essential to ensure that the NE did not undergo negative interactions such as coalescence, precipitation, or phase separation. The optimized NE demonstrated kinetic stability during stress studies involving high and low temperatures as well as high shear. Analysis using DSC and FT-IR showed that tacrolimus was evenly dispersed in NE formulations without interacting chemically with the ingredients or causing drug precipitation. The research indicated that incorporating linseed oil and fish oil into a tacrolimus NE gel could enhance the effectiveness of tacrolimus, resulting in reduced irritation and increased patient adherence due to its gel form (Mittal et al., 2021).

Zhang et al. developed tacrolimus-loaded cationic nanoemulsions (Tac-CNE) as a novel formulation to enhance the bioavailability of tacrolimus for immune-mediated inflammatory anterior ocular diseases (IIAODs) therapy by prolonging its precorneal residence time. Prepared through high-pressure homogenization and optimized through experiments. Tac-CNE exhibited a spherical morphology with a diameter of 178.8 nm and a zeta potential of +25.6 mV. In-vivo gamma scintigraphy studies demonstrated a significantly increased precorneal residence time for Tac-CNE compared to Tac-loaded neutral nanoemulsions (Tac-NE) and saline. Pharmacokinetic data in rabbits indicated that the relative bioavailability of Tac-CNE was higher than Tac-NE and marketed tacrolimus eye drops (Talymus®). The authors suggested that Tac-CNE showed accepted performance as a topical ophthalmic nanoformulation for the management of IIAODs (Zhang et al., 2020).

Wang et al. aimed to enhance the effectiveness of tacrolimus for treating dry eye disease by utilizing a cationic nanoemulsion (CNE) modified with thermosensitive in-situ gel (ISG) (CNE-ISG) for improved eye surface retention. CNE, consisting of water-in-oil nanoemulsions with cetalkonium chloride, was applied in this study. ISG is a transparent polymer solution that transforms into a gel upon application to the eye due to the phase change properties of the polymer, particularly poloxamers. Despite the potential of poloxamer 407 to extend drug release, its low adhesion activity led to the addition of adhesion-based polymers like hydroxypropyl methylcellulose and sodium hyaluronate in some ocular preparations. The use of thermosensitive in-situ-gel-modified cationic nanoemulsion as a tacrolimus delivery system demonstrated suitable administration conditions and prolonged immediate retention compared to other groups, suggesting its potential as an effective dosage form for dry eye disease treatment (Wang et al., 2023).

Although nanoemulsions offer various benefits, they also present several challenges for formulators. Key issues include identifying and comprehending the factors that contribute to variability in the development of nanoemulsions, which significantly influences the safety, therapeutic effectiveness, and stability of the drug product (Nishitani Yukuyama et al., 2017).

### 6.2 Nanocapsules

Nanocapsules are nano-sized vesicles composed of a polymeric shell that can encapsulate drugs within their core (Lima et al., 2022). Nanocapsules offer several significant advantages in various fields, particularly in drug delivery and biomedical applications. Their small size allows for enhanced penetration and absorption at the cellular level, improving the efficacy of therapies while minimizing systemic side effects. By encapsulating drugs in nanocapsules, it is possible to achieve targeted delivery to specific tissues or cells, thereby increasing therapeutic outcomes and reducing the required doses. Additionally, nanocapsules can provide controlled release of their payload, ensuring a sustained therapeutic effect over time and enhancing patient compliance. Their versatility also extends to encapsulating a wide range of substances, including hydrophobic drugs and sensitive biomolecules, thus broadening the scope of their applications in pharmaceuticals, cosmetics, and food science (Erdoğar et al., 2018).

Rebibo et al. worked on research about tacrolimus-loaded nanocapsules through solvent displacement for ophthalmic instillation. To prepare tacrolimus nanocapsules, firstly, poly (lactic-*co*-glycolic acid) was dissolved in acetone (organic phase). In the second step, castor oil with surfactant (Tween 80, Cremophor EL, or Lipoid E80,) was combined with organic phase. Tacrolimus was added to these compounds. The organic phase was gradually poured into the aqueous phase containing Solutol or PVA. To increase the stability of the colloidal dispersion, HPβCD as cryoprotectant was added to poly (lactic-co-glycolic acid) at a ratio of 1:10. The lyophilized and reconstituted colloidal nanocapsules were kept at 4 °C, 25 °C/60 % RH, and 40 °C/75 % RH for 3 months. The reconstituted samples were kept at 4 °C and 25 °C for 4 weeks. In the analysis of the physical and chemical properties of the nanocapsules, the key factors influencing how well the drug is encapsulated were found to be the utilized kind and amount of surfactant. The encapsulation efficiency was the lowest (61 %) with Lipoid E80 in the organic phase. The best formulation had 100 mg poly (lactic-co-glycolic acid), 10 mg tacrolimus, and 25 mg Tween 80 as a surfactant in the organic phase and 25 mg Solutol as an aqueous phase. Numerous factors, such as the type and concentration of cryoprotectant, affect the ability of nanocapsules to withstand lyophilization stress. The most favorable lyophilization outcomes were observed, with a poly (lactic-co-glycolic acid): HPβCD ratio of 1:10. After 3 months of storing the lyophilized formulation at 4, 25, and 40 °C, the concentration of the drug, size, and PDI remained almost the same as the original amount. The osmolality, average diameter, PDI, and content remained consistent over 14 days at 25 °C or 28 days at 4 °C, demonstrating the required ocular stability of the final reconstituted product (Rebibo et al., 2021).

Nanocapsules, while promising for various applications in drug delivery, diagnostics, and bioimaging, face several challenges that impede their widespread use. One primary issue is the difficulty in achieving uniformity in size and shape during the manufacturing process, which can affect their stability and functionality. Additionally, the interactions of nanocapsules with biological systems can lead to unpredictable behavior, including premature release of their contents or altered biodistribution, raising concerns about efficacy and safety (Ye and Chi, 2018).

### 6.3 Thin film freezing

Sahakijpijarn et al. developed a new inhalable formulation of tacrolimus using thin film freezing. Inhalation can maximize the delivery of tacrolimus to the targeted site, especially for preventing lung graft rejection with minimum systematic side effects. Furthermore, immunosuppressants are used as a treatment or prophylaxis for severe asthma that is resistant to typical treatments like corticosteroids. Minimizing the excipients in the pulmonary delivery of high-potency drugs is challenging because these excipients are vital for improving the dispersibility of micronized particles. New technologies like spray freeze drying, spray drying, and thin film freezing have been used in dry powder inhalations to produce porous particles and improve pharmaceutical efficacy. Thin film freezing is a precipitation method induced by freezing. This method can change the solubility of the drugs rapidly and particle formation follows supersaturation. In this study, 17 diverse formulations were prepared in terms of excipient type (lactose, mannitol, and trehalose), the solids content in the liquid (0.75 and 2.50 % *w*/*v*), process temperature (−70 and − 130 °C), and DL (50, 60, 70, 80, 90, 95, and 100 % *w*/w) in a mixture of acetonitrile and water (60:40 *v*/v) as shown in Table 6 (Sahakijpijarn et al., 2020).

**Table 6:** Thin film freezing formulations of tacrolimus.

Formulation number	Tacrolimus (% w/w)	Excipient type	Solid content (% w/v)	Temperature (°C)
1	50	Lactose	0.75	−70
2	60	Lactose	0.75	−70
3	70	Lactose	0.75	−70
4	80	Lactose	0.75	−70
5	90	Lactose	0.75	−70
6	95	Lactose	0.75	−70
7	100	–	0.75	−70
8	95	Mannitol	0.75	−70
9	95	Trehalose	0.75	−70
10	50	Lactose	0.75	−130
11	80	Lactose	0.75	−130
12	90	Lactose	0.75	−130
13	95	Lactose	0.75	−130
14	100	–	0.75	−130
15	50	Lactose	2.50	−70
16	95	Lactose	2.50	−70
17	95	Lactose	2.50	−130

**Fig. 5:**
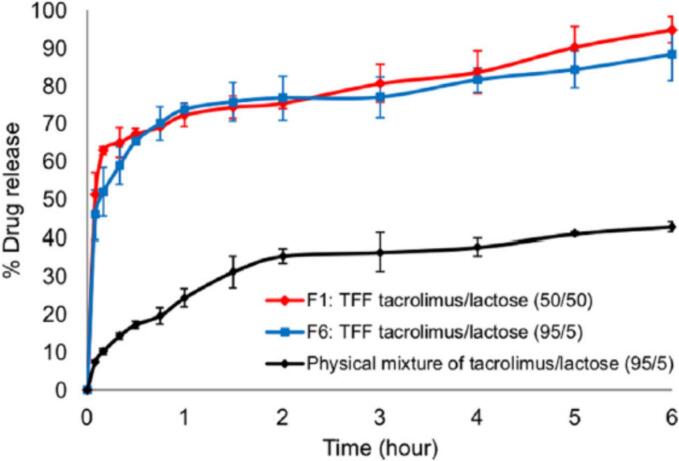
The dissolution of thin film freezing formulations 1 and 6 (Sahakijpijarn et al., 2020). Copyright [2020] [Elsevier].

The results of this investigation demonstrated that the solid content and the temperature were ineffective in the aerosol performance. The dissolution test of formulations 1 and 6 was compared to the physical mixture of tacrolimus and lactose. Both formulations were dissolved about 70 % in an hour and their dissolution was higher than the physical mixture (Fig. 5) (Sahakijpijarn et al., 2020).

The researchers selected formulations 1 and 6 for the stability test at 25 °C/60 % RH and 40 °C/75 % RH to determine physicochemical stability and aerosol performance. The XRD diffractograms exhibited a sharp peak in formulation 1 after a day of stress at 40 °C/75 % RH indicating that it had recrystallized. In contrast, formulation 6 was still amorphous for up to 6 months in several kinds of packaging. The stability tests of formulation 6 were carried out in 3 types of packaging including bulk powder with desiccant, encapsulated powder with desiccant, and encapsulated powder without desiccant. Then tacrolimus was quantified by the HPLC method. Moreover, particle size, moisture content, and specific surface area were analyzed for six months as shown in Table 7.

**Table 7:** Physicochemical properties of tacrolimus thin film freezing formulation with 95 % tacrolimus, 0.75 % w/v solid content, and lactose as an excipient in different stability tests.

Storage condition	Package	Time (month)	Tacrolimus (%)	Moisture content (%)	specific surface area (m^2^/g)	Particle size (μm)
25 °C/60 % RH	Encapsulated powder without desiccant	1	94.8	0.42	69.42	17.52
		3	94.2	0.54	68.13	19.68
		6	93.7	0.43	61.50	18.29
	Encapsulated powder with desiccant	1	93.6	0.28	73.42	18.53
		3	94.0	0.24	71.50	17.46
		6	95.4	0.32	63.61	17.52
	Bulk powder with desiccant	1	93.5	0.46	70.90	18.27
		3	93.9	0.40	68.99	18.52
		6	94.6	0.39	62.11	17.22
40 °C/75 % RH	Encapsulated powder without desiccant	1	93.6	1.43	15.76	8.74
		3	95.1	1.50	12.38	10.53
		6	94.3	1.54	9.92	8.64
	Encapsulated powder with desiccant	1	93.8	0.41	52.06	18.26
		3	94.1	0.33	52.67	17.25
		6	94.8	0.39	40.71	15.42
	Bulk powder with desiccant	1	93.6	0.56	29.52	17.81
		3	93.8	0.60	22.56	13.40
		6	94.2	0.62	19.79	11.21

The potency of tacrolimus did not change significantly after all of the storage conditions. Encapsulated powder without desiccant greatly altered particle size, moisture content, and specific surface area. In the presence of desiccant, encapsulated powder absorbed less than the bulk powder at both storage conditions. A rise in moisture levels corresponds to a reduction in specific surface area. In all packages, 40 °C/75 % RH caused a greater decline in specific surface area than 25 °C/60 % RH. Among different packaging, encapsulated powder without desiccant had the lowest specific surface area in both stability conditions. The particle size agreed with the alterations in specific surface area. Therefore, encapsulated powder with desiccant was chosen as the best package of thin film freezing formulation of tacrolimus (Sahakijpijarn et al., 2020).

Powders utilizing TFF technology demonstrate improved characteristics regarding morphology, solubility, stability, dissolution profile, and aerosol properties. The limitations of this technology are lower surface area/volume ratio and process stabilization (Pardeshi et al., 2022).

## 7 New additives and excipients

### 7.1 Polymers

Polymers are a versatile class of compounds that are commonly used in pharmaceutical drug formulations. These macromolecules are composed of repeating subunits called monomers, which can be manipulated to achieve specific properties such as biodegradability, controlled release of drugs, and biocompatibility (Sajjadi et al., 2023).

**Fig. 6:**
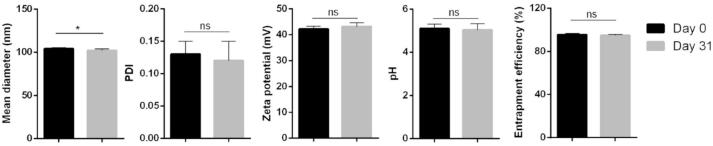
Variation in size (nm), PDI, zeta potential (mV), pH, and entrapment efficiency (EE %) of tacrolimus nanoparticles after 31 days at 25 °C. *: significantly different; ns: not significant (Castro et al., 2020). Copyright [2020] [Elsevier].

Castro et al. prepared tacrolimus-loaded nanoparticles through solvent (acetone) displacement and interfacial deposition of Eudragit RL100 as polymer. The stability of nanoparticles was evaluated after 31 days at 25 °C. The size, pH, zeta potential, PDI, and EE % were analyzed on days 0 and 31. The results demonstrated no significant alteration in zeta potential, pH, PDI, and EE %. Also, the size of nanoparticles decreased after 31 days (Fig. 6).

This alteration may be due to the dissolution of the polymeric shell but the hydrophobicity of tacrolimus inhibited the decrease in the amount of EE %. Tacrolimus was efficiently loaded into the nanoparticles and the formulation possessed good stability and appropriate physicochemical characterization for ocular application (Castro et al., 2020).

Mohammad et al. conducted a study to investigate how different formulation factors affect the development of tacrolimus-loaded lipid polymer hybrid nanoparticles (Tac-CS-LPHNs). The obtained results indicated that the ratio of 1,2-distearoyl-sn-glycero-3-phosphoethanolamine-N-[methoxy (polyethylene glycol)-2000 (DSPE-PEG 2000) to lecithin had a noticeable impact on particle size, with an increase in mass ratio leading to an increase in size but no change in zeta potential. Using DSPE instead of DSPE-PEG2000 and increasing polymer concentration also resulted in a significant elevation in particle size. Additionally, using acetonitrile as the solvent produced nanoparticles with better quality and smaller size compared to using acetone. The stability of these hybrid nanoparticles against changes in ionic strength, pH, storage temperature, and time, was also investigated. Short-term stability testing showed that the nanoparticles remained stable for up to 2 weeks after lyophilization at 25 °C or up to 8 months when stored at 4 °C as a lyophilized powder. However, aqueous dispersion was only stable for up to 3 weeks under the same storage conditions. Increasing ionic strength led to larger particle size and decreased zeta potential magnitude, with the zeta potential sometimes becoming positive. Changes in pH also affected zeta potential magnitude and particle size significantly (Mohammad and Ghareeb, 2021).

Camargo et al. conducted a study to assess the stability of colloidal suspensions of tacrolimus-loaded poly(ԑ-caprolactone) nanoparticles stored at 25 °C for 120 days. Various experiments including pH, particle size, PDI, zeta potential, and Raman spectroscopy were done. A significant decrease in pH was seen after 60 days for tacrolimus-loaded nanoparticles prepared at high drug concentrations (3 mg/mL). The loaded formulation showed significant pH changes after 90 days of storage. Non-loaded nanoparticles exhibited an increase in pH after 120 days. Particle size and PDI did not demonstrate noteworthy differences over the testing period. Zeta potential only had a statistically significant difference for tacrolimus-loaded nanoparticles after 120 days. Raman spectroscopy results indicated that the technique was effective in evaluating the stability of tacrolimus-loaded poly(ԑ-caprolactone) nanoparticles. The analysis revealed that degradation primarily occurred in amorphous regions of poly(ԑ-caprolactone), leading to enhanced polymer crystallinity. Overall, the tacrolimus-loaded nanoparticles demonstrated suitable stability within 60 days of preparation and could be confidently applied to skin disorders (Camargo et al., 2020).

In the context of tacrolimus formulations, various types of polymers have been explored to enhance the drug's stability, solubility, and bioavailability. In the current article, the various polymeric formulations that have been developed for tacrolimus delivery and their advantages and potential applications in the treatment of various diseases are comprehensively discussed:

Chitosan-based polymers have gained attention for their biocompatibility and bio-adhesive properties, making them suitable for encapsulating tacrolimus and facilitating its targeted delivery to specific sites within the body (Kaur et al., 2023). In a study, tacrolimus was encapsulated within a chitosan-based polymer through molecular envelope technology. After characterization of the formulation, the stability was studied under three storage conditions at room temperature (25 ± 2 °C / 60 ± 5 % RH), refrigerator (2–8 °C), and accelerated condition (40 ± 2 °C / 75 ± 5 % RH) for a month and size, pH, osmolarity, viscosity, and zeta potential were assessed on days 0, 7, and 30. As listed in Table 8, the formulation had good stability during refrigeration (Badr et al., 2021).

**Table 8:** Parameters determined during different storage conditions of the polymeric tacrolimus formulation.

Parameters	Tacrolimus concentration (mg/mL)			pH			Zeta potential (mV)			Osmolarity (mOsm/kg)			Viscosity (m.Pa.s)		
Storage condition	5 ± 3 °C	25 ± 2 °C 60 ± 5 %	40 ± 2 °C 75 ± 5 %	5 ± 3 °C	25 ± 2 °C 60 ± 5 %	40 ± 2 °C 75 ± 5 %	5 ± 3 °C	25 ± 2 °C 60 ± 5 %	40 ± 2 °C 75 ± 5 %	5 ± 3 °C	25 ± 2 °C 60 ± 5 %	40 ± 2 °C 75 ± 5 %	5 ± 3 °C	25 ± 2 °C 60 ± 5 %	40 ± 2 °C 75 ± 5 %
Day 0	0.99	0.99	0.99	7.1	7.1	7.1	+16	+16	+16	327	327	327	1.7	1.7	1.7
Day 7	1.00	0.87	0.27	6.8	6.7	6.8	+21	+21	+18	319	314	308	1.6	1.5	1.4
Day 30	0.94	0.41	0.03	6.9	6.5	6.3	+19	+24	+23	326	314	310	1.5	1.7	1.6

The issue of inadequate stability in chitosan-based systems limits their practical use, making it a significant challenge to achieve an adequate shelf life for chitosan formulations (Szymańska and Winnicka, 2015).

Fereig et al. developed chitosan nanoparticles through a modified solvent-free ionic gelation technique. To begin, tacrolimus was dissolved in a co-solvent (propylene glycol, PEG 400, or ethylene glycol) and heated at 60 °C while stirring for 10 min until complete dissolution. Penta‑sodium tripolyphosphate solution (TPP) was then added to the organic co-solvent solution at room temperature. This mixture was slowly injected into chitosan solutions of varying concentrations (dissolved in 1 % acetic acid and adjusted with NaOH) while stirring at room temperature. The suspension was left to stir for 30 min, then collected and physically characterized. The stability of the nanoparticles in a colloidal suspension was evaluated following three months of storage at 4 °C. Assessments were conducted to measure the size of particles, PDI, zeta potential, and EE %. Different pH values of chitosan solutions were tested in the preparation of tacrolimus nanoparticles. While pH values of 5 and 6 resulted in phase separation, pH 4 enabled the formation of nanoparticles. Chitosan solubility was found to decrease with higher pH values. To investigate the impact of chitosan:TPP:co-solvent ratio on tacrolimus nanoparticles, four different ratios were tested. The ratios included 5:2:3, 5:4:1, 5:3:2, and 5:1:4, with other variables being constant. The results showed that changing the ratio from 5:2:3 to 5:3:2 led to the production of smaller nanoparticles. This was attributed to the increased concentration of TPP, a polyanion, which decreased the molar ratio between amino and phosphate groups, resulting in more ionic interactions and smaller particle sizes. The precipitation of particles did not occur when the amount of TPP was increased to reach a ratio of 5:4:1 due to the low amount of co-solvent used and the saturation of chitosan crosslinking sites. This resulted in the excess drug precipitating. The ideal volume ratio for further studies was 5:2:3, balancing power savings and energy efficiency. This ratio was chosen based on the results obtained. To study the effect of different concentrations of chitosan on the particle size of nanoparticles, concentrations ranging from 0.1 % *w*/*v* to 2.5 % w/v were tested while keeping all other variables constant. The study found that increasing the concentration of chitosan solution resulted in a significant elevation in the particle size of the nanoparticles. This is because higher chitosan concentrations lead to a greater number of chains per unit volume, disrupting hydrogen bond attractions and electrostatic repulsions of different chitosan chains. This disruption ultimately leads to aggregation and the formation of larger particles.

The prepared formulations all had a positive charge between 17 and 33 mV, indicating strong repulsive forces between nanoparticles which prevent aggregation and maintain stability. Increasing drug concentration decreased zeta potential, possibly due to hydrogen bond formation between drug molecules and the cationic active sites of chitosan. Elevating chitosan concentration from 0.1 to 0.25 % led to a significant increase in zeta potential which could be correlated with the higher number of positively charged amino groups in chitosan. The impact of drug concentration on EE % of tacrolimus nanoparticles was studied using different concentrations of the drug and co-solvent. When comparing formulas with the same co-solvent concentration, increasing the drug concentration did not have a remarkable effect on EE or DL. This was likely due to the limited ability of the drug to interact with the hydrophilic polymer, causing saturation of the polymer matrix with a certain amount of drug. However, when comparing formulas with different co-solvent concentrations, increasing the drug concentration led to a significant reduction in EE. In summary, increasing the drug concentration did not have a notable effect on EE % when the co-solvent concentration was kept constant, but did decrease EE % when the co-solvent concentration was changed. The optimum formulation composed of 0.1 % chitosan solution, propylene glycol as co-solvent, 0.03 % tacrolimus, and chitosan:TPP:co-solvent ratio was 5:2:3. After 3 months of storage, there were minor, insignificant changes in the particle size and zeta potential of the optimum formulation, as expected due to the repulsive forces between particles preventing aggregation. However, there was a marked reduction in PDI likely due to larger particles settling. The EE % decreased by 12 % due to the low tendency of the hydrophobic drug to diffuse into the external medium (Fereig et al., 2021b).

Modi et al. worked on different batches of in-situ gel using the cold method described by Schmolka with slight modifications (Schmolka, 1972). Pluronic polymer was dissolved in cold water and stirred to obtain a clear solution, which was then refrigerated for at least 12 h. Chitosan gel was prepared separately and mixed with a pluronic solution. The concentration of tacrolimus was consistent in each batch, and the drug was added after dissolving it in methanol. The organic solvent was removed through evaporation, and the final volume of the formulation was adjusted to 30 mL with water. The stability of an in-situ gel formulation was tested by storing it at different temperatures (4 °C, room temperature, and 40 °C) for 90 days. The physical appearance, gelation time, gelation temperature, and drug content were monitored at regular intervals (30, 60, and 90 days) to evaluate the formulation and drug stability in the gel. Among thirteen tested formulations, only formulation with 22.5 g pluronic and 0.3 g chitosan met the criteria for gelation temperature and performance, such as forming a gel at 33.6 °C in the ophthalmic cavity and remaining as a liquid below 30 °C with fast gelation time and optimal spreadability. The stability study on the stored optimized formulation showed no significant changes in physical appearance, gelation temperature, gelation time, and drug content over 90 days at different temperatures. This confirmed that both the formulation and the drug remained stable during the study period (Modi et al., 2021).

### 7.2 Cyclodextrin

Cyclodextrins (CDs) are amphiphilic structures with a lipophilic center and hydrophilic outer layer with multiple hydroxyl groups. They are cyclic oligosaccharides bound together and can form complexes with drug molecules (Petitjean et al., 2021). Cyclodextrins offer many advantages, including low toxicity, effective bioavailability, and biodegradability. The main benefit of CDs is their ability to facilitate the interpretation of injectable formulations of poorly soluble drugs (Mohammad et al., 2020). They also allow for the inclusion of complexation and chemical modification, along with unique features like responsiveness to stimuli and the capacity to form precise aggregated nanostructures (Narayanan et al., 2022). These characteristics make cyclodextrins highly attractive for drug delivery applications requiring controlled and targeted release (Liu et al., 2021). Furthermore, they advance the physicochemical properties of drugs such as solubility, dissolution rate, stability, and bioavailability without molecular alterations. Natural CDs tend to self-assemble and form aggregates in aqueous media (Pandey, 2021).

**Fig. 7:**
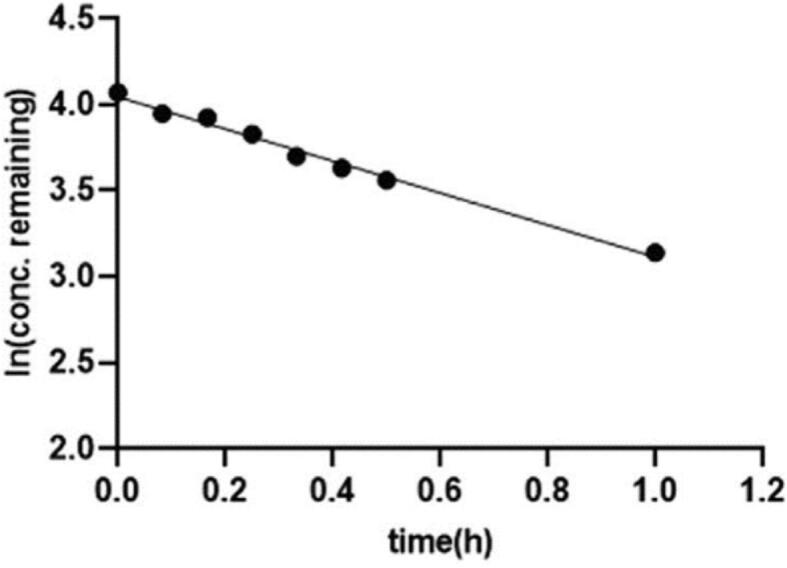
The plot of ln remaining tacrolimus concentration against time in 5 % 2-hydroxy-β-cyclodextrin (HPβCD) at pH 9 (Prajapati et al., 2020). Copyright [2020] [Elsevier].

**Fig. 8:**
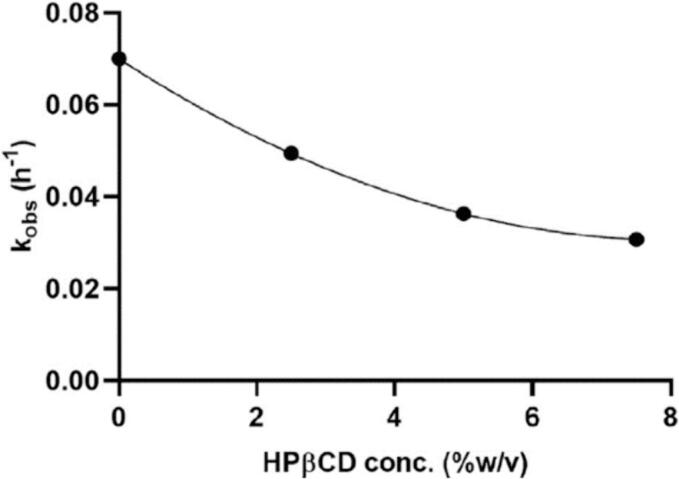
The effect of HPβCD concentration on the degradation rate constant in cyclodextrin solutions at pH 7.4 and 40 °C. The concentration of HPβCD was in the range of 0 to 7.5 % (Prajapati et al., 2020). Copyright [2020] [Elsevier].

Prajapati et al. studied the stability, solubility, and mechanism of tacrolimus degradation in cyclodextrin solutions (Prajapati et al., 2020). The kinetic study was performed at 40 °C with 2.48 mM of tacrolimus as the initial concentration. The solubility study was assessed by adding an excess amount of tacrolimus to different concentrations of cyclodextrin in aqueous solutions. Numerous cyclodextrins were tested. Tacrolimus could not form a complex with γ-cyclodextrin. The levels of first-order rate constant of tacrolimus degradation within the α- cyclodextrin and β-cyclodextrin complexes were less than 2-hydroxy-β-cyclodextrin (HPβCD). Therefore, HPβCD was the best stabilizer of the three tested cyclodextrins. The degradation of tacrolimus followed pseudo-first-order kinetics in HPβCD solutions at constant pH and temperature. The stability studies showed that tacrolimus was more stable at pH 5 and alkaline conditions accelerated its degradation. The main degradation pathways of tacrolimus in cyclodextrin solutions are hydrolysis of lactone and dehydration. Fig. 7 demonstrates a linear relationship between the natural logarithm of the percentage of the remaining tacrolimus concentration and time. As can be seen in Fig. 8, by increasing the concentration of HPβCD, the degradation rate of tacrolimus was decreased non-linearly. When the concentration of HPβCD was increased from 2.5 % to 5 %, the degradation rate reduced quickly but then leveled off at 7.5 %.

**Fig. 9:**
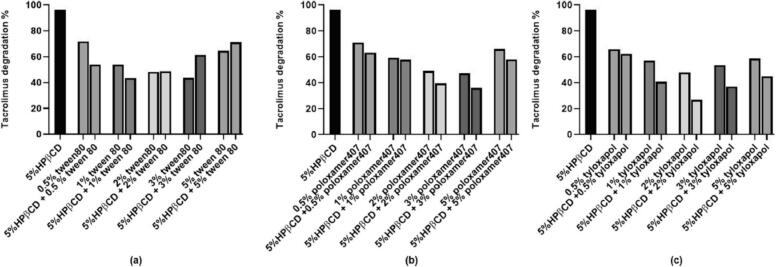
The percent of tacrolimus degradation after one cycle of autoclaving with 5 % w/v HPβCD and several concentrations of Tween 80 (a), poloxamer 407 (b), and tyloxapol (c) (Prajapati et al., 2020). Copyright [2020] [Elsevier].

Prajapati et al. used several surfactants such as poloxamer 407, tyloxapol, and Tween 80 to improve further the chemical stability of tacrolimus in cyclodextrins solutions. 0 to 5 % *w*/*v* surfactants were added to 5 % HPβCD solutions containing 100 μL of 2.48 mM tacrolimus stock solution and then the samples were exposed to one cycle of autoclaving. The concentration of tacrolimus was evaluated by the UHPLC method. Fig. 9a revealed that tacrolimus was more stable at 5 % HPβCD solution containing 1 % Tween 80. Solutions containing only Tween 80 in concentrations of more than 2 % were more stable than the combination of Tween 80 and HPβCD. Up to 3 % poloxamer 407, decreased tacrolimus degradation either when only poloxamer or a combination of cyclodextrin and poloxamer were utilized (Fig. 9b). Fig. 9c displays tacrolimus degradation in tyloxapol solutions. Tacrolimus concentration was maximum at 2 % tyloxapol then it decreased in solutions containing equal or more than 3 % tyloxapol. The surfactants protected tacrolimus from degradation through micelle formation.

**Fig. 10:**
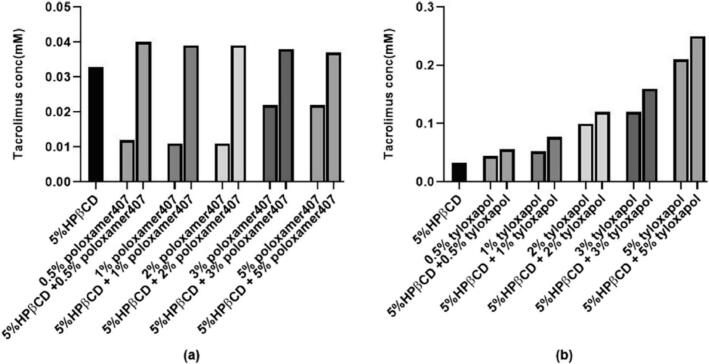
Solubility tests of tacrolimus in 5 % HPβCD with poloxamer 407 (a) and tyloxapol (b) (Prajapati et al., 2020). Copyright [2020] [Elsevier].

Poloxamer 407 and tyloxapol were used in the solubility test as remarked in Fig. 10a The obtained results proved that poloxamer 407 can increase the solubility of tacrolimus in an independent manner of the concentration. The main reason for this condition is the competition influence of tacrolimus and poloxamer 407 for the cavity of HPβCD. Fig. 10b revealed that tacrolimus solubility increased by elevating the tyloxapol concentration due to tacrolimus wettability and micelle formation.

Garcia-Otero et al. worked on developing an eye drop using HPβCD (García-Otero et al., 2021). The solubility study was carried out by adding an excess amount of tacrolimus to various concentrations of HPβCD. These samples were shaken in a shaker at 25 °C and 250 rpm for a week. Then, they were centrifuged for half an hour. The supernatant was diluted with water in a ratio of 1:5. To assess the vehicle effect on the solubility test, three different HPβCD solutions (20 %, 30 %, and 40 % *w*/*v*) were diluted 1:5 with balanced salt solution (BSS) and Liquifilm® besides water. Liquifilm® is an artificial tear containing 1.4 % polyvinyl alcohol (PVA) and benzalkonium chloride. Subsequently, tacrolimus concentration was determined by the HPLC method. The formulations were inclusive of three main parts: HPβCD, tacrolimus, and vehicle as illustrated in Table 9.

**Table 9:** The compositions of tacrolimus eye drops with cyclodextrin.

Formulation		1	2	3	4
Composition	HPβCD (% w/v)	20	20	40	40
	Tacrolimus (% w/v)	0.01	0.01	0.02	0.02
	Vehicle	Balanced salt solution	Liquifilm®	Balanced salt solution	Liquifilm®

The physicochemical properties of the formulations were evaluated by determining pH, osmolarity, surface tension, and squeezing force. The stability test was carried out with formulations containing more amounts of tacrolimus numbered 3 and 4 in Table 9. The samples were kept at three storage conditions for 4 months: 4 ± 2 °C, 25 ± 2 °C, and 40 ± 2 °C. The amount of tacrolimus, pH, and osmolarity was analyzed on days 0, 15, 30, 45, 78, and 120. The microbiological stability was performed by adding 1 mL of each formulation to three culture media: Sabouraud-chloramphenicol agar, fluid thioglycate medium, and blood agar. The samples were grown at 37 °C and the microbial growth was determined on days 2, 10, and 15, respectively. The solubility test results demonstrated that the concentration of tacrolimus was increased by adding more HPβCD.

Also, the effect of BBS and Liquifilm® was studied. There were no significant differences between 20 % and 30 % HPβCD in all vehicles but these solutions demonstrated remarkable differences compared to 40 % in all the vehicles. Therefore, in future studies only 20 % and 40 % HPβCD were utilized. Significant differences were observed between Liquifilm® and BSS. The possible reason is the attribution of Liquifilm® in the formation of the ternary complex between tacrolimus, PVA, and HPβCD that increases solubility by synergistic solubilization.

The resulting data from the physicochemical characterization of the formulations are summarized in Table 10. All formulations met the pH value described for eye drops (pH 4 to 8). Nevertheless, the osmolarity quantities of the formulations were higher than the limit value for ophthalmic administration (1220.5 mOsm/kg) which can induce inflammation. The ideal surface tension for eye drops is similar to the tear fluid (42–46 mN/m). The formulations had a greater amount of surface tension than tears. The squeezing force is affected by the viscosity and surface tension of the formulation and the design of the dropper tip. Consequently, the same type of packaging was used in this test. There was no meaningful difference between these four formulations.

**Table 10:** Physicochemical parameters of tacrolimus/HPβCD formulations.

Formulation	1	2	3	4
pH	7.036	6.968	7.203	6.933
Osmolarity	359.3	283.6	628	383
Surface tension	54.63	58.46	51.5	58.43

The stability data from formulations 3 and 4 proved that temperature was an important factor affecting their tacrolimus concentration. The formulations kept at 40 ± 2 °C were unstable in the first week. The degradation rate of formulation 3 was more than 4 for this temperature. In the formulations stored at room temperature on day 15, the concentration of tacrolimus was less than 90 % of the initial amount. However, the formulations were stable for 3 months at 4 °C. In formulation 4, Liquifilm was utilized as a vehicle that was beneficial to the stability of the formulation due to the presence of benzalkonium chloride.

Osmolarity and pH measurements displayed no significant changes over 3 months of all storage conditions. The microbiological stability indicated no presence of microorganisms and no macroscopic alterations such as color change, precipitation, and turbidity throughout the study. In this study, the best formulation based on the estimation of pharmacokinetic parameters was formulation 4 with 0.011 (min ^−1^) of rate constant (K), 86.22 min of the half-life (t_1/2_), 123.31 % × min of AUC_0_^∞^, 90.51 min of mean residence time, and 78.82 % of % remaining formulation at 75 min (García-Otero et al., 2021).

Tao et al. utilized Soluplus, a copolymer of polyvinyl caprolactam, polyvinyl acetate, and polyethylene glycol, to enhance the stability and absorption of tacrolimus in a supersaturated state. The inclusion complex (Tac-CD) was prepared using dimethyl-β-cyclodextrin (DM-β-CD) and displayed favorable dissolution profiles. However, the drug concentration decreased rapidly in supersaturated conditions, reaching 10.64 ± 0.69 μg/mL of tacrolimus at 12 h. The ternary complex (Tac-SCD) incorporating Soluplus maintained a steadier drug concentration. The addition of 1.2 % Soluplus in Tac-SCD significantly improved the supersaturated stability of the inclusion complex, resulting in 62.90 ± 3.34 μg/mL of tacrolimus at 12 h. Soluplus was also effective in reducing the crystallization and degradation of tacrolimus during stress testing. These findings suggested that Soluplus was highly effective in enhancing the supersaturated stability of the tacrolimus inclusion complex (Tao et al., 2019).

Soe et al. worked on research to enhance the aqueous solubility of tacrolimus by using HPβCD for complexation. The researchers also aimed to create new eye drop formulations by incorporating tacrolimus into Zein nanoparticles (ZnNP) or NE stabilized by ZnNP, with or without HPβCD. All four tacrolimus formulations displayed favorable physicochemical characteristics and remained physically stable in simulated tear fluid for at least 2 h. However, the tacrolimus/HPβCD-loaded NE stabilized by ZnNP (TAC/CD-NE) exhibited the highest EE % and mucoadhesive properties among the formulations. Moreover, TAC/CD-NE maintained its physical and chemical stability for up to three months when kept refrigerated. Overall, the study findings demonstrated that TAC/CD-loaded NE stabilized by ZnNP provided safe and effective delivery of tacrolimus, showing promise as an eye drop formulation for treating retinal diseases (Soe et al., 2023).

## 8 Future research prospects

Tacrolimus is an immunosuppressant that prevents transplant rejection and treats several dermatology and ocular disorders. So, the need for various dosage forms of tacrolimus like ophthalmic drops, topical formulations, and oral administration is incredible. There are some key areas of future research for developing new tacrolimus formulations. Researchers can explore novel drug delivery using new techniques such as nanotechnology, micelles, liposomes, and polymers.

To overcome drug-excipient incompatibility issues in preformulation studies, further research can be conducted to understand the interactions between tacrolimus and excipients. This could involve computational modeling like Design-Expert and simulation studies to predict potential drug-excipient interactions and optimize formulation design. Isothermal stress tests and using the PharmDE database can be suggested to study the compatibility of tacrolimus with new excipients like cyclodextrins, thermal gel-forming polymers, and excipient matrices for 3D printing (Gupta et al., 2019) (Wang et al., 2021c).

Improvement in the stability and solubility of tacrolimus is crucial in developing the formulations. Future research can focus on developing accelerated stability testing methods that can accurately predict the shelf-life of tacrolimus formulations. By understanding the degradation pathways of tacrolimus under different storage conditions, researchers can design more robust formulations that maintain drug stability over extended periods. Also, with the advancements in analytical techniques, researchers can develop more sensitive and accurate methods for analyzing tacrolimus and detecting potential impurities. Mass spectrometry, nuclear magnetic resonance (NMR), and surface analysis methods can be explored.

## 9 Conclusion

In conclusion, tacrolimus faces various physicochemical challenges such as thermal instability, photostability, low solubility, and drug-excipient incompatibility. These challenges can affect the efficacy and stability of tacrolimus in various formulations. However, analytical methods such as thermal, spectroscopic, microscopic, and HPLC have been developed to overcome these challenges and ensure the quality of tacrolimus. Additionally, solutions such as lipid-based nanocarriers and polymers have been found to enhance the physicochemical stability of tacrolimus in different formulations. Overall, developing and implementing these analytical methods and solutions play a crucial role in ensuring their stability and efficacy in various formulations, ultimately improving patient outcomes and medication efficiency. Further research and innovation in this area are needed to address the evolving challenges in the formulation and stability of tacrolimus.

## Funding

This research did not receive any specific grant from funding agencies in the public, commercial, or not-for-profit sectors.

## Institutional review board statement

The study was approved by the research ethics committee of Tabriz University of Medical Sciences (IR.TBZMED.PHARMACY.REC.1402.042).

## Informed consent statement

Not applicable.

## CRediT authorship contribution statement

**Sara Sajjadi:** Writing – original draft. **Ali Shayanfar:** Writing – review & editing, Project administration. **Farhad Kiafar:** Supervision. **Mohammadreza Siahi-Shadbad:** Supervision.

## Declaration of competing interest

The authors declare no conflict of interest.

## Data Availability

Not applicable.

## References

[bb0005] Albaghdadi A.J., Coyle K., Kan F.W. (2022). Low-dose tacrolimus promotes the migration and invasion and nitric oxide production in the human-derived first trimester extravillous trophoblast cells in vitro. Int. J. Mol. Sci..

[bb0010] Ali R., Farah A., Binkhathlan Z. (2017). Development and characterization of methoxy poly(ethylene oxide)-block-poly(ε-caprolactone) (PEO-b-PCL) micelles as vehicles for the solubilization and delivery of tacrolimus. Saudi Pharm. J..

[bb0015] Alsaad A.A., Hussien A.A., Gareeb M.M. (2020). Solid lipid nanoparticles (SLN) as a novel drug delivery system: a theoretical review. Syst. Rev. Pharm..

[bb0020] Al-Tamimi D.J., Hussein A.A. (2021). Formulation and characterization of self-microemulsifying drug delivery system of tacrolimus. Iraqi J. Pharm. Sci..

[bb0025] Andrea B., Osvaldo B., Samer H. (2022). Topical tacrolimus for the treatment of external eye inflammation in children. Expert. Rev. Ophthalmol..

[bb0030] Andrews L.M., Li Y., De Winter B.C., Shi Y.-Y., Baan C.C., Van Gelder T., Hesselink D.A. (2017). Pharmacokinetic considerations related to therapeutic drug monitoring of tacrolimus in kidney transplant patients. Expert Opin. Drug Metab. Toxicol..

[bb0035] Annett S., Moore G., Robson T. (2020). FK506 binding proteins and inflammation related signalling pathways; basic biology, current status and future prospects for pharmacological intervention. Pharmacol. Therapeut..

[bb0040] Arora C.J., Rafiq M., Shumack S., Gupta M. (2020). The efficacy and safety of tacrolimus as mono-and adjunctive therapy for vitiligo: a systematic review of randomised clinical trials. Aust. J. Dermatol..

[bb0045] Badr M.Y., Abdulrahman N.S., Schatzlein A.G., Uchegbu I.F. (2021). A polymeric aqueous tacrolimus formulation for topical ocular delivery. Int. J. Pharm..

[bb0050] Barrieu M., Chennell P., Yessaad M., Bouattour Y., Wasiak M., Jouannet M., Le Basle Y., Sautou V. (2022). Physicochemical stability of a novel tacrolimus ophthalmic formulation for the treatment of ophthalmic inflammatory diseases. Pharmaceutics.

[bb0055] Bhalani D.V., Nutan B., Kumar A., Singh Chandel A.K. (2022). Bioavailability enhancement techniques for poorly aqueous soluble drugs and therapeutics. Biomedicines.

[bb0060] Böer T.M., Procópio J.V.V., Nascimento T.G.D., Macêdo R.O. (2013). Correlation of thermal analysis and pyrolysis coupled to GC–MS in the characterization of tacrolimus. J. Pharm. Biomed. Anal..

[bb0065] Bressán I.G., Giménez M.I., Llesuy S.F. (2021). Validation of a simple liquid chromatography coupled to tandem mass spectrometry method for the simultaneous determination of tacrolimus, sirolimus, everolimus and cyclosporin A in dried matrix on paper discs. J. Mass Spectrom. Adv. Clin. Lab.

[bb0070] Camargo G.D.A., Costa Filha A.R.C.D., Lyra A.M., Novatski A., Nadal J.M., de Lara L.S., Dias D.T., Nascimento E.A.D., Rocha Silva U., Jacinto C., Farago P.V. (2020). Stability testing of tacrolimus-loaded poly(ԑ-caprolactone) nanoparticles by physicochemical assays and Raman spectroscopy. Vib. Spectrosc..

[bb0075] Castro B.F.M., de Oliveira Fulgêncio G., Domingos L.C., Cotta O.A.L., Silva-Cunha A., Fialho S.L. (2020). Positively charged polymeric nanoparticles improve ocular penetration of tacrolimus after topical administration. J. Drug Deliv. Sci. Technol..

[bb0080] Chettri A., Subba A., Singh G.P., Bag P.P. (2024). Pharmaceutical co-crystals: a green way to enhance drug stability and solubility for improved therapeutic efficacy. J. Pharm. Pharmacol..

[bb0085] Dasineh S., Akbarian M., Ebrahimi H.A., Behbudi G. (2021). Tacrolimus-loaded chitosan-coated nanostructured lipid carriers: preparation, optimization and physicochemical characterization. Appl. Nanosci..

[bb0090] Elumalai K., Srinivasan S., Shanmugam A. (2024). Review of the efficacy of nanoparticle-based drug delivery systems for cancer treatment. Biomed. Tech..

[bb0095] Erdoğar N., Akkın S., Bilensoy E. (2018). Nanocapsules for drug delivery: an updated review of the last decade. Recent Pat. Drug Deliv. Formul..

[bb0100] Ezquer-Garin C., Ferriols-Lisart R., Alós-Almiñana M. (2017). Stability of tacrolimus ophthalmic solution. Am. J. Health Syst. Pharm..

[bb0105] Fereig S.A., El-Zaafarany G.M., Arafa M.G., Abdel-Mottaleb M.M. (2021). Self-assembled tacrolimus-loaded lecithin-chitosan hybrid nanoparticles for in vivo management of psoriasis. Int. J. Pharm..

[bb0110] Fereig S.A., El-Zaafarany G.M., Arafa M.G., Abdel-Mottaleb M.M. (2021). Tacrolimus-loaded chitosan nanoparticles for enhanced skin deposition and management of plaque psoriasis. Carbohydr. Polym..

[bb0115] Fonseca L.A., Bertan A.S., Cremasco M.A. (2021). Group Contribution as selection criteria for nutrients fermentation media in the production of tacrolimus. Chem. Eng. Trans..

[bb0120] García-Otero X., Díaz-Tomé V., Varela-Fernández R., Martín-Pastor M., González-Barcia M., Blanco-Méndez J., Mondelo-García C., Bermudez M.A., Gonzalez F., Aguiar P. (2021). Development and characterization of a tacrolimus/hydroxypropyl-β-cyclodextrin eye drop. Pharmaceutics.

[bb0125] Garg A., Garg R. (2022). Current advances in colloidal based delivery systems for Tacrolimus. J. Drug Deliv. Sci. Technol..

[bb0130] Ghasemiyeh P., Mohammadi-Samani S. (2018). Solid lipid nanoparticles and nanostructured lipid carriers as novel drug delivery systems: applications, advantages and disadvantages. Res. Pharm. Sci..

[bb0135] Gomes G.S., Frank L.A., Contri R.V., Longhi M.S., Pohlmann A.R., Guterres S.S. (2023). Nanotechnology-based alternatives for the topical delivery of immunosuppressive agents in psoriasis. Int. J. Pharm..

[bb0140] Gote V., Mandal A., Alshamrani M., Pal D. (2020). Self-assembling tacrolimus nanomicelles for retinal drug delivery. Pharmaceutics.

[bb0145] Gupta K.R., Pounikar A.R., Umekar M.J. (2019). Drug excipient compatibility testing protocols and charaterization: a review. Asian J. Chem. Sci..

[bb0150] Ha J.-M., Kang S.-Y., Park C.-W., Bin S.-A., Rhee Y.-S., Seo J.-W., Kim S.-H., Chi S.-C., Park E.-S. (2012). Effect of poloxamer on physicochemical properties of tacrolimus solid dispersion improving water solubility and dissolution rate. J. Pharm. Investig..

[bb0155] Hamed Y., Elsayed M., Essawy A., Hamed M. (2023). Topical administration of tacrolimus and systemic corticosteroids in treatment of oral and nasal mucous membrane pemphigoid in a sample of Egyptian population: a randomized control Clinical trial. Egypt. Dent. J..

[bb0160] Hazari S.A., Kaur H., Karwasra R., Abourehab M.A., Khan A.A., Kesharwani P. (2023). An overview of topical lipid-based and polymer-based nanocarriers for treatment of psoriasis. Int. J. Pharm..

[bb0165] Hirasawa W., Sei S., Mineda M., Endo N., Matahira Y., Makino K., Tsukada R., Sato H., Onoue S. (2020). UniORV, a new multi-unit dosage form, improved biopharmaceutical properties of tacrolimus in rats and humans. Pharm. Res..

[bb0170] Hirota A., Shoji J., Inada N., Shiraki Y., Yamagami S. (2022). Evaluation of clinical efficacy and safety of prolonged treatment of vernal and atopic keratoconjunctivitis using topical tacrolimus. Cornea.

[bb0175] Jadhav Bharat V., Tattu Arpita B., Jadhav Supriya B. (2024). Review on self micro emulsifying drug delivery system SMEDDS. Int. J. Pharm. Sci..

[bb0180] Jain S., Addan R., Kushwah V., Harde H., Mahajan R.R. (2019). Comparative assessment of efficacy and safety potential of multifarious lipid based Tacrolimus loaded nanoformulations. Int. J. Pharm..

[bb0185] K B. (2018). Lipid nano particulate drug delivery: an overview of the emerging trend. Pharma Innov. J..

[bb0190] Kailasam V., Cheruvu S.S., Malani M., Kameswari S.M.S., Kesharwani P., Nirmal J. (2022). Recent advances in novel formulation approaches for tacrolimus delivery in treatment of various ocular diseases. J. Drug Deliv. Sci. Technol..

[bb0195] Kamelnia R., Ahmadi-Hamedani M., Kamelnia E., Darroudi M. (2024). Enhancing insulin stability via efficacy chemical modifications: a comprehensive review. Int. J. Pharm..

[bb0200] Kaneko Y., Kawahito Y., Kojima M., Nakayama T., Hirata S., Kishimoto M., Endo H., Seto Y., Ito H., Nishida K. (2021). Efficacy and safety of tacrolimus in patients with rheumatoid arthritis–a systematic review and meta-analysis. Mod. Rheumatol..

[bb0205] Kang J.-H., Chon J., Kim Y.-I., Lee H.-J., Oh D.-W., Lee H.-G., Han C.-S., Kim D.-W., Park C.-W. (2019). Preparation and evaluation of tacrolimus-loaded thermosensitive solid lipid nanoparticles for improved dermal distribution. Int. J. Nanomedicine.

[bb0210] Kaur M., Sharma A., Puri V., Aggarwal G., Maman P., Huanbutta K., Nagpal M., Sangnim T. (2023). Chitosan-based polymer blends for drug delivery systems. Polymers.

[bb0215] Khan A., Alsahli M.A., Aljasir M.A., Maswadeh H., Mobark M.A., Azam F., Allemailem K.S., Alrumaihi F., Alhumaydhi F.A., Alwashmi A.S. (2022). Safety, stability, and therapeutic efficacy of long-circulating TQ-incorporated liposomes: implication in the treatment of lung cancer. Pharmaceutics.

[bb0220] Khan A.S., Shah K.U., Mohaini M.A., Alsalman A.J., Hawaj M.A.A., Alhashem Y.N., Ghazanfar S., Khan K.A., Niazi Z.R., Farid A. (2022). Tacrolimus-loaded solid lipid nanoparticle gel: Formulation development and in vitro assessment for topical applications. Gels.

[bb0225] Kovačević A.B., Müller R.H., Keck C.M. (2020). Formulation development of lipid nanoparticles: improved lipid screening and development of tacrolimus loaded nanostructured lipid carriers (NLC). Int. J. Pharm..

[bb0230] Lima A.L., Gratieri T., Cunha-Filho M., Gelfuso G.M. (2022). Polymeric nanocapsules: a review on design and production methods for pharmaceutical purpose. Methods.

[bb0235] Liu Z., Ye L., Xi J., Wang J., Feng Z.-G. (2021). Cyclodextrin polymers: Structure, synthesis, and use as drug carriers. Prog. Polym. Sci..

[bb0240] Liu W., Pan W., Zou M., Jin S., Mi R., Cheng G., Piao H. (2023). Tacrolimus and paclitaxel co-loaded O/O ointment without surfactant: Synergistic combinations for the treatment of psoriasis. Eur. J. Pharm. Biopharm..

[bb0245] Llinàs-Mallol L., Redondo-Pachón D., Raïch-Regué D., Pérez-Sáez M.J., Yélamos J., Duran X., Faura A., López-Botet M., Pascual J., Crespo M. (2020). Long-term redistribution of peripheral lymphocyte subpopulations after switching from calcineurin to mTOR inhibitors in kidney transplant recipients. J. Clin. Med..

[bb0250] Luis J., Alsaedi A., Phatak S., Kapoor B., Rees A., Westcott M. (2022). Efficacy of tacrolimus in uveitis, and the usefulness of serum tacrolimus levels in predicting disease control. Results from a single large center. Ocul. Immunol. Inflamm..

[bb0255] Lv X., Qi J., Zhou M., Shi B., Cai C., Tang Y., Pan T., Han Y. (2020). Comparative efficacy of 20 graft-versus-host disease prophylaxis therapies for patients after hematopoietic stem-cell transplantation: a multiple-treatments network meta-analysis. Crit. Rev. Oncol. Hematol..

[bb0260] Malode M.G.P., Chauhan S.A., Bartare S.A., Malode L.M., Manwar J.V., Bakal R.L. (2021). A critical review on nanoemulsion: Advantages, techniques and characterization. J. Appl. Pharm. Sci. Res..

[bb0265] Mandal S., Maharana P.K., Kaweri L., Asif M.I., Nagpal R., Sharma N. (2023). Management and prevention of corneal graft rejection. Indian J. Ophthalmol..

[bb0270] Mansoori M.J.A. (2023). SMEDDS: emerging technique for enhancement of drug solubility and bioavailability. J. Surv. Fish Sci..

[bb0275] Mika A., Stepnowski P. (2016). Current methods of the analysis of immunosuppressive agents in clinical materials: a review. J. Pharm. Biomed. Anal..

[bb0280] Mittal S., Ali J., Baboota S. (2021). Enhanced anti-psoriatic activity of tacrolimus loaded nanoemulsion gel via omega 3-Fatty acid (EPA and DHA) rich oils-fish oil and linseed oil. J. Drug Deliv. Sci. Technol..

[bb0285] Modi D., Mohammad, Warsi M.H., Garg V., Bhatia M., Kesharwani P., Jain G.K. (2021). Formulation development, optimization, and in vitro assessment of thermoresponsive ophthalmic pluronic F127-chitosan in situ tacrolimus gel. J. Biomater. Sci. Polym. Ed..

[bb0290] Mohammad A., Ghareeb M. (2021). Tacrolimus monohydrate loaded lipid polymer hybrid nanoparticles Formulation and stability study. Kerbala J. Pharm. Sci..

[bb0295] Mohammad A., Singh S., Swain S. (2020). Cyclodextrins: concept to applications, regulatory issues and challenges. Nanomed. Res. J..

[bb0300] Namiki Y., Fujiwara A., Kihara N., Koda S., Hane K., Yasuda T. (1995). Determination of the immunosuppressive drug tacrolimus in its dosage forms by high-performance liquid chromatography. Chromatographia.

[bb0305] Narayanan G., Shen J., Matai I., Sachdev A., Boy R., Tonelli A.E. (2022). Cyclodextrin-based nanostructures. Prog. Mater. Sci..

[bb0310] Nishitani Yukuyama M., Tomiko Myiake Kato E., Lobenberg R., Araci Bou-Chacra N. (2017). Challenges and future prospects of nanoemulsion as a drug delivery system. Curr. Pharm. Des..

[bb0315] Oellerich M., Armstrong V.W., Schütz E., Shaw L.M. (1998). Therapeutic drug monitoring of cyclosporine and tacrolimus. Clin. Biochem..

[bb0320] Ong S.C., Gaston R.S. (2021). Thirty years of tacrolimus in clinical practice. Transplantation.

[bb0325] Pandey A. (2021). Cyclodextrin-based nanoparticles for pharmaceutical applications: a review. Environ. Chem. Lett..

[bb0330] Pardeshi S.R., Kole E.B., Kapare H.S., Chandankar S.M., Shinde P.J., Boisa G.S., Salgaonkar S.S., Giram P.S., More M.P., Kolimi P. (2022). Progress on thin film freezing technology for dry powder inhalation formulations. Pharmaceutics.

[bb0335] Patel P., Patel H., Panchal S., Mehta T. (2012). Formulation strategies for drug delivery of tacrolimus: an overview. Int. J. Pharm. Investig..

[bb0340] Patel P., Ahir K., Patel V., Manani L., Patel C. (2015). Drug-Excipient compatibility studies: first step for dosage form development. Pharm. Innov..

[bb0345] Peltonen L., Strachan C.J. (2020). Degrees of order: a comparison of nanocrystal and amorphous solids for poorly soluble drugs. Int. J. Pharm..

[bb0350] Peterka T.R., Hren J., Bastarda A., Bergles J., Urleb U. (2015). Solid state compatibility study and characterization of a novel degradation product of tacrolimus in formulation. J. Pharm. Biomed. Anal..

[bb0355] Petitjean M., García-Zubiri I.X., Isasi J.R. (2021). History of cyclodextrin-based polymers in food and pharmacy: a review. Environ. Chem. Lett..

[bb0360] Ponnammal P., Kanaujia P., Yani Y., Ng W.K., Tan R.B.H. (2018). Orally disintegrating tablets containing melt extruded amorphous solid dispersion of tacrolimus for dissolution enhancement. Pharmaceutics.

[bb0365] Prajapati M., Eiriksson F.F., Loftsson T. (2020). Stability characterization, kinetics and mechanism of tacrolimus degradation in cyclodextrin solutions. Int. J. Pharm..

[bb0370] Radhakrishnan A., Kuppusamy G., Ponnusankar S., Mutalik S. (2021). Towards next-generation personalization of tacrolimus treatment: a review on advanced diagnostic and therapeutic approaches. Pharmacogenomics.

[bb0375] Rahman Z., Zidan A., Khan M.A. (2013). Tacrolimus: Effectiveness, Safety and Drug Interactions.

[bb0380] Rajpoot K., Tekade M., Pandey V., Nagaraja S., Youngren-Ortiz S.R., Tekade R.K. (2020). Drug Delivery Systems.

[bb0385] Ravanshad Y., Azarfar A., Ravanshad S., Kalat M.N., Ghasemi A., Golsorkhi M., Mostafavian Z., Esmaeeli M., Majd H.M. (2020). A comparison between tacrolimus and Cyclosporine as immunosuppression after renal transplantation in children, a meta-analysis and systematic review. Iran. J. Kidney Dis..

[bb0390] Rebibo L., Tam C., Sun Y., Shoshani E., Badihi A., Nassar T., Benita S. (2021). Topical tacrolimus nanocapsules eye drops for therapeutic effect enhancement in both anterior and posterior ocular inflammation models. J. Control. Release.

[bb0395] Rodriguez-Rodriguez A.E., Porrini E., Torres A. (2021). Beta-cell dysfunction induced by tacrolimus: a way to explain type 2 diabetes?. Int. J. Mol. Sci..

[bb0400] Rozman Peterka T., Trdan Lušin T., Bergles J., Ham Z., Grahek R., Urleb U. (2019). Forced degradation of tacrolimus and the development of a UHPLC method for impurities determination. Acta Pharma..

[bb0405] Saatkamp R.H., Dos Santos B.M., Sanches M.P., Conte J., Rauber G.S., Caon T., Parize A.L. (2023). Drug-excipient compatibility studies in formulation development: a case study with benznidazole and monoglycerides. J. Pharm. Biomed. Anal..

[bb0410] Sahakijpijarn S., Moon C., Ma X., Su Y., Koleng J.J., Dolocan A., Williams R.O. (2020). Using thin film freezing to minimize excipients in inhalable tacrolimus dry powder formulations. Int. J. Pharm..

[bb0415] Sajjadi S., Siahi-Shadbad M., Afshar Mogaddam M.R. (2022). Stability tests and analytical methods of tacrolimus: a review. IA.

[bb0420] Sajjadi S., Gholizadeh-Hashjin A., Shafizadeh F., Marefat S., Hamidi S., Farjami A. (2023). Advancing biomedicine with gel-based materials and composites: a comprehensive review. J. Appl. Polym. Sci..

[bb0425] Sallustio B.C. (2010). LC–MS/MS for immunosuppressant therapeutic drug monitoring. Bioanalysis.

[bb0430] Savić V., Ilić T., Nikolić I., Marković B., Čalija B., Cekić N., Savić S. (2019). Tacrolimus-loaded lecithin-based nanostructured lipid carrier and nanoemulsion with propylene glycol monocaprylate as a liquid lipid: Formulation characterization and assessment of dermal delivery compared to referent ointment. Int. J. Pharm..

[bb0435] Schmolka I.R. (1972). Artificial skin I. Preparation and properties of pluronic F-127 gels for treatment of burns. J. Biomed. Mater. Res..

[bb0440] Shi Q., Li J., Ding F. (2012). Development and validation of method for the determination of related substances of tacrolimus in tacrolimus capsules and degradation studies. Int. J. ChemTech Res..

[bb0445] Singh A., Narsinghani T. (2023). An extensive comprehension of forced degradation studies of new drug substances and products. J. Coast. Life Med..

[bb0450] Skytte D.M., Jaroszewski J.W., Johansen K.T., Hansen S.H., Hansen L., Nielsen P.G., Frydenvang K. (2013). Some transformations of tacrolimus, an immunosuppressive drug. Eur. J. Pharm. Sci..

[bb0455] Soe H.M.S.H., Maw P.D., Asasutjarit R., Loftsson T., Jansook P. (2023). Tacrolimus/hydroxypropyl-β-cyclodextrin-loaded nanoemulsions stabilized by Zein-Soluplus® nanoparticles for retinal diseases. J. Drug Deliv. Sci. Technol..

[bb0460] Soni H., Sharma S. (2021). Current update on nanoemulsion: a review. Sch. Int. J. Anat. Physiol..

[bb0465] Sun K., Hu K. (2021). Preparation and characterization of tacrolimus-loaded SLNs in situ gel for ocular drug delivery for the treatment of immune conjunctivitis. Drug Des. Dev. Ther..

[bb0470] Szymańska E., Winnicka K. (2015). Stability of chitosan—a challenge for pharmaceutical and biomedical applications. Mar. Drugs.

[bb0475] Tao C., Huo T., Zhang Q., Song H. (2019). Effect of Soluplus on the supersaturation and absorption of tacrolimus formulated as inclusion complex with dimethyl-β-cyclodextrin. Pharm. Dev. Technol..

[bb0480] Tao C., Huo T., Zhang M., Chen Z., Zhang X., Song H. (2020). Evaluation of the stability and absorption of tacrolimus self-microemulsifying drug delivery system. J. Drug Deliv. Sci. Technol..

[bb0485] Umar B.U., Rahman S., Dutta S., Islam T., Nusrat N., Chowdhury K., Ahmad W.F.S.B.W., Haque M., Umar B.U.U., Fakuradzi W.F.S.W.A. (2022). Management of atopic dermatitis: The role of tacrolimus. Cureus.

[bb0490] Viegas J.S.R., Praça F.G., Caron A.L., Suzuki I., Silvestrini A.V.P., Medina W.S.G., Del Ciampo J.O., Kravicz M., Bentley M.V.L.B. (2020). Nanostructured lipid carrier co-delivering tacrolimus and TNF-α siRNA as an innovate approach to psoriasis. Drug Deliv. Transl. Res..

[bb0495] Wang D., Cheow W.S., Amalina N., Faiezin M., Hadinoto K. (2021). Selecting optimal pharmaceutical excipient formulation from life cycle assessment perspectives: a case study on ibuprofen tablet formulations. J. Clean. Prod..

[bb0500] Wang N., Sun H., Dong J., Ouyang D. (2021). PharmDE: a new expert system for drug-excipient compatibility evaluation. Int. J. Pharm..

[bb0505] Wang N., Sun H., Dong J., Ouyang D. (2021). PharmDE: a new expert system for drug-excipient compatibility evaluation. Int. J. Pharm..

[bb0510] Wang Q., Wu Z., Wang F., Zhang H., Gan L. (2023). Tacrolimus-loaded cationic nanoemulsion in-situ gel system: in-vitro characterization and performance in a dry-eye rabbit model. J. Pharm. Sci..

[bb0515] Wathoni N., Suhandi C., Elamin K.M., Lesmana R., Hasan N., Mohammed A.F.A., El-Rayyes A., Wilar G. (2024). Advancements and challenges of nanostructured lipid carriers for wound healing applications. Int. J. Nanomedicine.

[bb0520] Yan B., Ma Y., Guo J., Wang Y. (2020). Self-microemulsifying delivery system for improving bioavailability of water insoluble drugs. J. Nanopart. Res..

[bb0525] Yan L., Zhang Z., Zhang Y., Yang H., Qiu G., Wang D., Lian Y. (2021). Improvement of tacrolimus production in Streptomyces tsukubaensis by mutagenesis and optimization of fermentation medium using Plackett–Burman design combined with response surface methodology. Biotechnol. Lett..

[bb0530] Ye C., Chi H. (2018). A review of recent progress in drug and protein encapsulation: approaches, applications and challenges. Mater. Sci. Eng. C.

[bb0535] Zelesky T., Baertschi S.W., Foti C., Allain L.R., Hostyn S., Franca J.R., Li Y., Marden S., Mohan S., Ultramari M. (2023). Pharmaceutical forced degradation (stress testing) endpoints: a scientific rationale and industry perspective. J. Pharm. Sci..

[bb0540] Zhang J., Liu Z., Tao C., Lin X., Zhang M., Zeng L., Chen X., Song H. (2020). Cationic nanoemulsions with prolonged retention time as promising carriers for ophthalmic delivery of tacrolimus. Eur. J. Pharm. Sci..

[bb0545] Zhao M., He F., Yang Y., Lin W., Qiu W., Meng Q., Zhang J., Zhou Z. (2022). Therapeutic efficacy of tacrolimus in vernal keratoconjunctivitis: a meta-analysis of randomised controlled trials. Eur. J. Hosp. Pharm..

[bb0550] Zheng B., McClements D.J. (2020). Formulation of more efficacious curcumin delivery systems using colloid science: enhanced solubility, stability, and bioavailability. Molecules.

[bb0555] Zolgharnein J., Goudarzy F., Ghasemi J.B. (2024). A novel g-C3N4@La:Y2O3 nanocomposite fluorescence sensor for simultaneous determination and photocatalytic degradation of Ulipristal acetate and Tacrolimus using Doehlert design optimization. J. Photochem. Photobiol. A Chem..

